# Exercise and Pain Neuroscience Education for Patients With Chronic Pain After Total Knee Arthroplasty: A Randomized Clinical Trial

**DOI:** 10.1001/jamanetworkopen.2024.12179

**Published:** 2024-05-24

**Authors:** Jesper B. Larsen, Søren T. Skou, Mogens Laursen, Niels Henrik Bruun, Lars Arendt-Nielsen, Pascal Madeleine

**Affiliations:** 1 Musculoskeletal Health and Implementation, Department of Health Science and Technology, Faculty of Medicine, Aalborg University, Aalborg, Denmark; 2 Research Unit for Musculoskeletal Function and Physiotherapy, Department of Sports Science and Clinical Biomechanics, University of Southern Denmark, Odense, Denmark; 3 The Research and Implementation Unit PROgrez, Department of Physiotherapy and Occupational Therapy, Næstved-Slagelse-Ringsted Hospitals, Region Zealand, Denmark; 4 Orthopedic Surgery Research Unit, Aalborg University Hospital, Aalborg, Denmark; 5 Research Data and Biostatistics, Aalborg University Hospital, Aalborg, Denmark; 6 Translational Pain Biomarkers, Department of Health Science and Technology, Faculty of Medicine, Aalborg University, Aalborg, Denmark; 7 ExerciseTech, Department of Health Science and Technology, Faculty of Medicine, Aalborg University, Aalborg, Denmark

## Abstract

**Importance:**

Up to 20% of patients develop chronic pain after total knee arthroplasty (TKA), yet there is a scarcity of effective interventions for this population.

**Objective:**

To evaluate whether neuromuscular exercise and pain neuroscience education were superior to pain neuroscience education alone for patients with chronic pain after TKA.

**Design, Setting, and Participants:**

A superiority randomized clinical trial was conducted at 3 outpatient clinics at Aalborg University Hospital in Denmark. Participants with moderate-to-severe average daily pain intensity and no signs of prosthesis failure at least 1 year after primary TKA were included. Participant recruitment was initiated on April 12, 2019, and completed on October 31, 2022. The 12-month follow-up was completed on March 21, 2023.

**Interventions:**

The study included 24 sessions of supervised neuromuscular exercise (2 sessions per week for 12 weeks) and 2 total sessions of pain neuroscience education (6 weeks between each session) or the same pain neuroscience education sessions alone. The interventions were delivered in groups of 2 to 4 participants.

**Main Outcomes and Measures:**

The primary outcome was change from baseline to 12 months using the mean score of the Knee Injury and Osteoarthritis Outcome Score, covering the 4 subscales pain, symptoms, activity of daily living, and knee-related quality of life (KOOS_4_; scores range from 0 to 100, with higher scores indicating better outcomes). The outcome assessors and statistician were blinded. All randomized participants were included in the intention-to-treat analysis.

**Results:**

Among the 69 participants (median age, 67.2 years [IQR, 61.2-71.9 years]; 40 female [58%]) included in the study, 36 were randomly assigned to the neuromuscular exercise and pain neuroscience education group, and 33 to the pain neuroscience education–alone group. The intention-to-treat analysis showed no between-group difference in change from baseline to 12 months for the KOOS_4_ (7.46 [95% CI, 3.04-11.89] vs 8.65 [95% CI, 4.67-12.63] points; mean difference, −1.33 [95% CI, −7.59 to 4.92]; *P* = .68). Among the 46 participants who participated in the 12-month assessment in the 2 groups, 16 (34.8%) experienced a clinically important improvement (a difference of ≥10 points on the KOOS_4_) with no between-group difference. No serious adverse events were observed.

**Conclusions and Relevance:**

In this randomized clinical trial, the results demonstrated that neuromuscular exercises and pain neuroscience education were not superior to pain neuroscience education alone in participants with chronic pain after TKA. Approximately one-third of the participants, regardless of intervention, experienced clinically important improvements. Future studies should investigate which patient characteristics indicate a favorable response to exercises and/or pain neuroscience education.

**Trial Registration:**

ClinicalTrials.gov Identifier: NCT03886259

## Key Points

**Question:**

What is the effect of neuromuscular exercise and pain neuroscience education compared with pain neuroscience education alone on pain and function in patients with chronic pain for more than 1 year after total knee arthroplasty?

**Findings:**

In this randomized clinical trial of 69 patients, neuromuscular exercise and pain neuroscience education did not provide superior pain and function outcomes compared with pain neuroscience education alone, although approximately one-third of all patients experienced clinically important improvements.

**Meaning:**

Findings from this study suggest that neuromuscular exercise and pain neuroscience education do not provide superior pain and function outcomes compared with pain neuroscience education alone, but clinically important improvements in pain and function can be elicited in patients with chronic pain after total knee arthroplasty.

## Teaser

This randomized clinical trial investigates the effect of neuromuscular exercise and pain neuroscience education vs pain neuroscience education alone on pain and function among patients in Denmark with chronic pain for more than 1 year after total knee arthroplasty.

## Introduction

End-stage knee osteoarthritis is commonly treated with total knee arthroplasty (TKA).^1^ In 2018, more than 715 000 TKAs were performed in the US,^2^ and the number is expected to rise to 1.9 million annually by 2030.^3^ Most patients undergoing TKA surgery will experience a positive outcome in terms of pain relief and improved functional performance, but 15% to 20% of patients will develop chronic pain after TKA.^4,5^ Chronic pain after TKA is defined as pain present for at least 3 to 6 months following surgery.^6^

Patients have described the chronic pain after TKA as extreme, constant, and requiring maximal effort to endure.^7^ Furthermore, activities of daily living (eg, walking and stair climbing) are impaired in patients with chronic pain after TKA when compared with patients with knee osteoarthritis prior to surgery.^8^

Chronic pain after TKA is considered multifactorial and can be influenced by physiological factors, such as central pain mechanisms, and psychosocial factors.^6,8^ There is a scarcity of high-quality evidence and guidelines on effective treatments of chronic pain after TKA.^6,9^ The lack of evidence-based treatment guidelines leads to inadequate access to optimal treatment and the risk of patients feeling abandoned by the health care system.^10^

Studies have evaluated the inclusion of early postoperative exercises to avoid patients developing chronic pain after TKA but have not found this approach effective.^11,12^ However, a combination of exercise and education treatment modalities could induce beneficial treatment effects in patients with chronic pain after TKA,^13^ but to our knowledge, this has never been investigated.

Therefore, we conducted a superiority randomized clinical trial with the purpose of investigating whether a 12-week treatment consisting of neuromuscular exercise and pain neuroscience education (PNE) would prove superior in terms of improving pain and function compared with receiving PNE alone. It was hypothesized that the participants randomized to neuromuscular exercise and PNE would improve significantly more from baseline to 12 months compared with participants randomized to PNE alone.

## Methods

### Study Design

**Figure 1.**
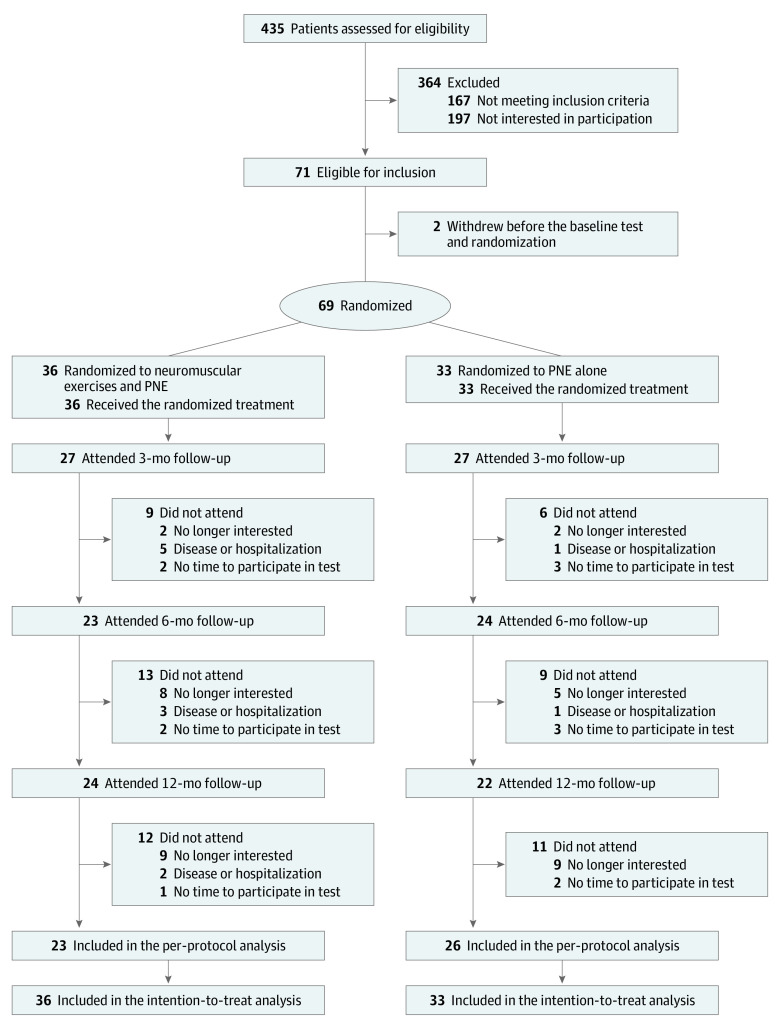
Patient Flow Diagram From Baseline to the 12-Month Follow-up PNE indicates pain neuroscience education.

The study was designed as a parallel-group superiority randomized clinical trial, entitled the NEPNEP (Neuromuscular Exercises and Pain Neuroscience Education for Chronic Pain) trial. An open access study protocol was published to ensure research quality and transparency.^14^ The trial followed the Consolidated Standards of Reporting Trials (CONSORT) reporting guideline for randomized clinical trials.^15^ The patient flow diagram is provided as Figure 1, and the trial protocol is provided in Supplement 1. The CONSORT, Template for Intervention Description and Replication (TIDieR), and Consensus on Exercise Reporting Template (CERT) checklists are provided in eAppendices 1-3, respectively, in Supplement 2. The trial was approved by the North Denmark Region Committee on Health Research Ethics. All participants signed informed consent before inclusion in the trial.

### Participants

Participants were recruited from Aalborg University Hospital (Aalborg, Denmark), which included 3 hospital sites in Farsoe, Thisted, and Aalborg. The hospital research database was used to identify participants who underwent TKA at least 1 year before recruitment. Eligible participants were contacted by mail and telephone and invited to participate in the study. Participants willing to enroll and meeting the eligibility criteria of primary TKA due to knee osteoarthritis 12 months or longer after their surgery and, in the index knee, chronic pain for longer than 6 months and an average daily pain score of 4 or more (moderate to severe pain) on a numeric rating scale (ranging from 0 to 10, in which 0 is no pain, and 10 is maximum pain) over the last week were included. The major exclusion criteria were chronic pain due to loosening of an implant or a prosthesis failure requiring revision surgery or primary pain area other than the index knee (eg, low back pain or upper extremity pain). A full list of eligibility criteria can be found in the study protocol (Supplement 1).^14^ Participants received the interventions at 1 of the 3 outpatient clinics at Aalborg University Hospital (Farsoe, Thisted, and Aalborg) dependent on their geographical preferences and on which day and time for exercise and PNE suited them best. Recruitment was initiated on April 12, 2019, and completed on October 31, 2022. The 12-month follow-up was completed on March 21, 2023.

### Patient and Public Involvement

Two patients with chronic pain after TKA assisted in designing the trial from a patient perspective. The patients gave feedback concerning study procedures, interventions, and outcome measures and how to describe and explain the study in layperson’s terms to possible participants.

### Randomization and Masking

The participants were randomized in a 1:1 ratio and allocated to 1 of 2 intervention arms, neuromuscular exercises and PNE or PNE alone. Randomization with treatment group concealment was done by the project manager (J.B.L.) by using computer-generated random numbers in permuted blocks of 4 to 8 participants. Outcome assessment was performed by trained outcome assessors (not involved in the study), who were masked toward treatment allocation. The statistician (N.H.B.) conducting the analysis was masked toward group allocation.

### Interventions

The neuromuscular exercises and PNE group received a 12-week neuromuscular exercise program^16^ and PNE. The neuromuscular exercise program has previously been found feasible for patients following TKA surgery.^17^ One-hour group-based sessions consisting of 2 to 4 participants were held twice a week (24 sessions in total). Sessions were supervised by trained physiotherapists and included individualization of the exercise difficulty considering each participant’s physical ability and pain intensity. Full details of the neuromuscular exercise program can be found in the study protocol (Supplement 1).^14^

The PNE consisted of two 1-hour group-based educational sessions. The first session was held before the first exercise session for the neuromuscular exercise and PNE group, and the second session took place 6 weeks later. A physiotherapist trained in PNE (J.B.L.) delivered the sessions to both groups. Both intervention groups received the same content in the PNE sessions. The overall aim of PNE was to change maladaptive pain cognitions, enabling the participants to reconceptualize their pain^18^ and thereby engage in self-management of their symptoms. Following both PNE sessions, a short information leaflet, summarizing the PNE topics, was given to the participants. Content for the PNE sessions can be found in eMethods 1 and 2 in Supplement 2. Assessments of outcomes were conducted at baseline and at 3, 6, and 12 months.

### Outcomes

#### Primary Outcome

The primary outcome was prespecified and reported in the study protocol^14^ and the statistical analysis plan.^19^ The primary outcome was the between-group change from baseline to 12 months for the Knee Injury and Osteoarthritis Outcome Score (KOOS), using the mean score of the 4 subscales: pain, symptoms, activities of daily living, and knee-related quality of life (KOOS_4_). The subscales, which include a fifth dimension—sport and recreation—are scored on a 5-point Likert scale; the total is converted into a range of 0 (worst) to 100 (best).^20,21^ A prespecified minimum clinically important difference of 10 points was used to indicate whether a clinically relevant between-group improvement from baseline to the 12-month follow-up had occurred.^22^ The KOOS questionnaire has shown validity, reliability, and responsiveness as a patient-reported outcome measure following TKA.^23^

#### Secondary Outcomes

Six prespecified secondary outcomes were evaluated as between-group changes using the mean difference from baseline to a 12-month follow-up.^19^ All 5 KOOS subscales, including the sport and recreation subscale, were reported individually to support the clinical interpretation of the primary outcome.^22^ The overall change in a participant’s knee condition was measured using the global perceived effect scale by their answer to the question: “How are your knee problems now compared with before you entered this study?” The global perceived effect scale was administered on a 7-point Likert scale ranging from 1 (improved, an important improvement) to 7 (worse, an important worsening). The global perceived effect scale has shown excellent reliability.^24^ Three physical performance tests were included.^25^ The time to complete the 40-m fast-paced walk test and the stair-climb test, a test of ascending and descending 9 steps on a staircase, was recorded. For the 30-second chair-stand test, the maximum number of chair-rise repetitions within 30 seconds was registered.^25^ The physical performance tests have been found reliable.^26,27^ Use of pain medication was evaluated by asking participants whether they had used pain medication over last week (yes or no). Adverse events occurring during the trial period were registered as either serious or nonserious events by participant self-report and/or by the physiotherapists supervising the neuromuscular exercises. Serious adverse events were defined according to the definitions from the US Food and Drug Administration, and nonserious adverse events comprised all other events.^28^ Other treatments initiated because of the index knee received during the trial period were registered by self-reporting from the participants.

### Statistical Analysis

A statistical analysis plan was published and available before the 12-month follow-up, and any analyses were initiated.^19^ The analyses were conducted as predefined in the statistical analysis plan. To avoid the risk of misleading interpretation, the results from the intention-to-treat analysis were presented to the author group in a blinded version (coded as group A and group B). In writing, the authors agreed on 2 separate interpretations of the results,^29^ and documentation for the interpretations was registered online.^30^ After finalizing the interpretations, the randomization code was broken, and the appropriate interpretation was chosen.

#### Sample Size Calculation

For this superiority randomized clinical trial, a sample-size calculation was conducted to estimate the sample size required to detect a between-group minimum clinically important difference in change of 10 points from baseline to the 12-month follow-up for the KOOS_4_ (with an SD of 15).^17,22^ The calculation revealed that 49 participants were required in both groups to achieve a study power of 90% from baseline to the 12-month follow-up for the between-group comparison, using a 2-sided significance level of .05. To account for a possible loss to follow-up of 20%, a total of 60 participants in each group were planned to be enrolled. However, the trial was impacted by the COVID-19 pandemic, making recruitment particularly difficult and causing a higher dropout rate than anticipated. Therefore, we were not able to recruit the preplanned number of participants and decided to stop recruitment after recruiting for 42 months.

#### Data Analysis

The main analysis consisted of the between-group differences in mean change from baseline to the 12-month follow-up. Analysis of all outcomes was performed according to the intention-to-treat principle. Furthermore, a prespecified per-protocol analysis was conducted, including participants who participated in at least 18 of 24 (75%) neuromuscular exercise sessions and participated in both PNE sessions (valid for both groups).

Data were checked for normal distribution by reviewing data frequency in histograms and tests for normality (Shapiro-Wilk). Based on the observations, median and IQR were recorded. For the primary and secondary outcomes (except use of pain medication), repeated measures mixed-effects models were applied, with participants as the random effect and time for visit (baseline and 3, 6, and 12 months) and treatment arm (neuromuscular exercises and PNE or PNE alone) as fixed effects, with adjustment for baseline imbalance. Interaction between follow-up and treatment arm was also included in the models. Two models are reported: model 1, adjusted for participant, follow-up, treatment arm, and interaction between follow-up and treatment arm; model 2 further included adjustment for age, sex, and body mass index. The between-group comparison for use of pain medication within the last week was dichotomized as yes or no, and relative risks were analyzed using a Poisson regression model with robust error variance. No analysis for difference in adverse events was required because no adverse events were registered in the PNE-alone group.

A prespecified responder analysis was conducted to illustrate the proportion of participants in the 2 intervention groups who experienced a minimum clinically important difference of at least 10 points in KOOS_4_. The proportions were compared using a χ^2^ test.

For all outcomes, 95% CIs are presented. A 95% CI, including 10 points or more for the primary outcome, KOOS_4_, was interpreted as a clinically meaningful difference.^22^ A 2-sided *P* < .05 was considered significant. All analyses were performed in Stata, version 18 (StataCorp LLC).

## Results

A total of 69 patients (median age, 67.2 years [IQR, 61.2-71.9 years]; 40 female [58%]) and 29 male [42%]) were recruited. Overall, 435 patients were assessed for eligibility (Figure 1). Of these, 364 were excluded, leaving 71 eligible for inclusion; 2 patients withdrew before randomization. Thirty-six participants were randomized to receive neuromuscular exercises and PNE and 33 participants to receive PNE alone. The participants’ baseline characteristics were comparable (Table 1).^31^ The mean body mass index in our population was greater than 33 (calculated as weight in kilograms divided by height in meters squared), most participants had at least 1 comorbidity, and there was a group-average score in the Hospital Anxiety and Depression Scale^31^ that indicated clinical depression.

**Table 1.** Patient Baseline Characteristics

Characteristics	Neuromuscular exercises and PNE group (n = 36)	PNE-alone group (n = 33)
Age, median (IQR), y	68.8 (62.7-72.9)	65.8 (60.1-71.3)
Sex, No. (%)		
Female	22 (61)	18 (55)
Male	14 (39)	15 (45)
Height, median (IQR), cm	1.7 (1.6-1.8)	1.7 (1.6-1.8)
Body mass, median (IQR), kg	93.0 (76.7-103.7)	95.1 (80.2-108.8)
Body mass index, median (IQR)^a^	33.1 (27.7-36.1)	33.3 (29.5-36.0)
Average daily pain intensity over the last week, median (IQR)^b^	6.0 (5.0-7.0)	5.0 (4.0-6.0)
Right index knee, No. (%)	17 (47)	16 (49)
Right dominant leg, No. (%)	30 (86)	30 (91)
Time since surgery, median (IQR), y	3.2 (1.8-4.9)	2.7 (1.7-4.3)
Total knee arthroplasty in nonindex knee, No. (%)	7 (19)	11 (33)
One or more comorbidities, No. (%)	31 (86)	28 (85)
Distribution of comorbidities, No. (%)		
Osteoarthritis in joints other than the index knee	20 (56)	18 (55)
Chronic pain from sites other than the index knee	22 (61)	19 (58)
Chronic obstructive pulmonary disease	4 (11)	2 (6)
Diabetes	5 (14)	3 (9)
Cardiovascular disease	4 (11)	6 (18)
Hospital Anxiety and Depression Scale–Anxiety total score, median (IQR)^c^	8.0 (5.2-9.8)	7.0 (3.0-9.0)
Hospital Anxiety and Depression Scale–Depression total score, median (IQR)^c^	12.5 (6.0-15.0)	13.0 (4.5-15.0)

Abbreviation: PNE, pain neuroscience education.

^a^ Calculated as weight in kilograms divided by height in meters squared.

^b^ Eligibility included a pain score of 4 or more (moderate to severe pain) on a numeric rating scale, ranging from 0 to 10, with the higher score indicating maximum pain.

^c^ Scores range from 0 to 21: 0 to 7, no case; 8 to 10, borderline case; and 11 and above, case.^31^

All participants were included in the intention-to-treat analysis. Twenty-three participants (64%) in the neuromuscular exercises and PNE group and 26 (79%) in the PNE-alone group adhered to the intervention and were included in the per-protocol analysis. The completion rates for the 12-month follow-up assessment were 24 of 36 participants (67%) for the neuromuscular exercises and PNE group and 22 of 33 (67%) for the PNE-alone group. Dropout reasons are reported in Figure 1. The baseline characteristics for the participants adhering to the 12-month assessment and the participants lost to follow-up were comparable (eTable 1 in Supplement 2).

**Figure 2.**
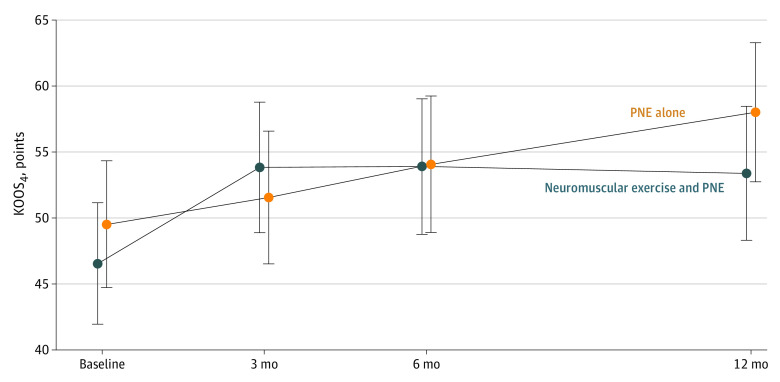
Changes in the 4 Knee Injury and Osteoarthritis Outcome Score Subscales (KOOS_4_) The KOOS_4_ primary outcome includes the subscales pain, symptoms, function of daily living, and knee-related quality of life; scores range from 0 to 100, with higher scores indicating better outcomes. Data points are means; error bars represent 95% CI. PNE indicates pain neuroscience education.

The intention-to-treat analysis showed no between-group difference in improvement from baseline to the 12-month follow-up for the primary outcome KOOS_4_, illustrated by an adjusted mean difference of −1.33 (95% CI, −7.59 to 4.92; *P* = .68) (Figure 2). Both groups experienced significant improvements in KOOS_4_ from baseline to the 12-month follow-up, with the neuromuscular exercise and PNE group improving 7.46 points (95% CI, 3.04-11.89; *P* = .001) and the PNE-alone group improving 8.65 points (95% CI, 4.67-12.63; *P* < .001) (Table 2).

**Table 2.** Intention-to-Treat Analysis for the Primary and Secondary Outcomes for Mean Change From Baseline to the 12-Month Follow-Up

Outcome	Improvement in neuromuscular exercise and PNE group, mean (95% CI)^a^	Improvement in PNE-alone group, mean (95% CI)^b^	Between-group adjusted difference		
			Model 1, mean (95% CI)^c^	Model 2, mean (95% CI)^d^	*P* values for model 2
**Primary outcome**					
KOOS_4_^e^	7.46 (3.04 to 11.89)	8.65 (4.67 to 12.63)	−1.19 (−7.14 to 4.76)	−1.33 (−7.59 to 4.92)	.68
**Secondary outcomes**					
KOOS subscales					
Pain	6.41 (1.51 to 11.30)	9.85 (5.82 to 13.87)	−3.44 (−9.77 to 2.90)	−4.02 (−10.85 to 2.82)	.25
Symptoms	6.96 (0.04 to 13.87)	8.23 (1.86 to 14.59)	−1.27 (−10.67 to 8.13)	−1.62 (−11.50 to 8.26)	.75
Activities of daily living	4.49 (0.19 to 8.80)	7.66 (1.83 to 13.49)	−3.16 (−10.41 to 4.08)	−3.66 (−10.79 to 3.47)	.31
Sport and recreation	5.06 (−0.08 to 10.19)	9.41 (3.05 to 15.77)	−4.35 (−12.52 to 3.83)	−5.40 (−13.25 to 2.46)	.18
Knee-related quality of life	10.60 (4.11 to 17.09)	9.79 (3.64 to 15.94)	0.81 (−8.13 to 9.75)	1.81 (−6.86 to 10.48)	.68
Global perceived effect scale^f^	2.82 (2.24 to 3.39)	2.80 (2.27 to 3.33)	0.02 (−0.77 to 0.80)	0.02 (−0.82 to 0.86)	.96
Time to walk 40 m, s	−3.11 (−5.66 to −0.56)	−1.68 (−4.65 to 1.28)	−1.43 (−5.34 to 2.48)	−0.97 (−5.19 to 3.25)	.65
Time to complete stair-climb test, s	−2.53 (−4.73 to −0.33)	−1.99 (−3.54 to −0.43)	−0.55 (−3.24 to 2.15)	−0.42 (−3.26 to 2.43)	.77
30-s Chair-stand repetitions	0.93 (0.22 to 1.63)	1.88 (0.96 to 2.79)	−0.95 (−2.11 to 0.21)	−1.05 (−2.26 to 0.16)	.09

Abbreviations: KOOS, Knee Injury and Osteoarthritis Outcome Score; KOOS_4_, KOOS subscales including pain, symptoms, activity of daily living, and knee-related quality of life; PNE, pain neuroscience education.

^a^ There were 144 possible data points (36 at baseline and at 3, 6, and 12 months), except for the global perceived effect, which had 108 possible data points (36 at at 3, 6, and 12 months).

^b^ There were 132 possible data points (33 at baseline and at 3, 6, and 12 months), except for the global perceived effect, which had 99 possible data points (33 at 3, 6, and 12 months).

^c^ Model 1 adjusted for patient, follow-up, treatment arm, and interaction between follow-up and treatment arm.

^d^ Model 2 followed model 1 and further included adjustment for age, sex, and body mass index.

^e^ Scores range from 0 to 100, with higher scores indicating better outcomes.

^f^ Scores are based on a 7-point Likert scale ranging from 1 (improved, an important improvement) to 7 (worse, an important worsening).

**Figure 3.**
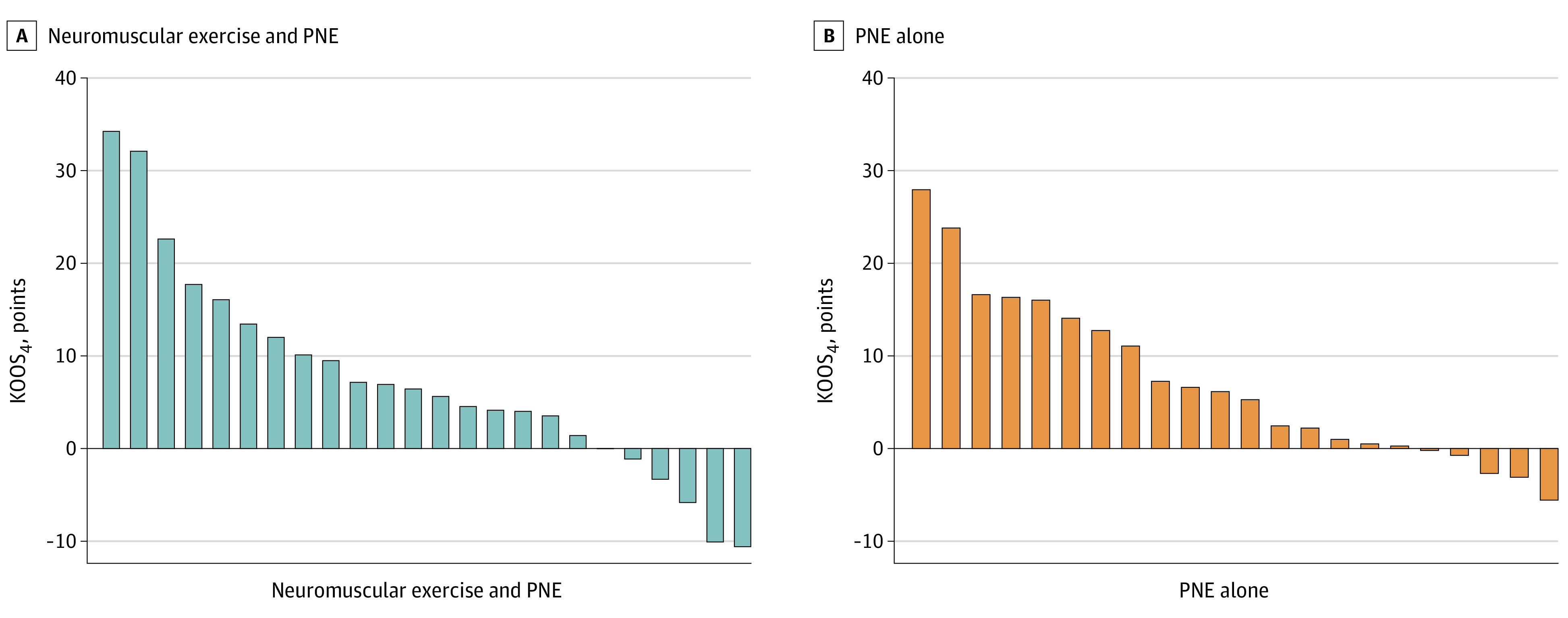
Individual Changes in the 4 Knee Injury and Osteoarthritis Outcome Score Subscales (KOOS_4_) From Baseline to the 12-Month Follow-up The KOOS_4_ primary outcome includes the subscales pain, symptoms, function of daily living, and knee-related quality of life; scores range from 0 to 100, with higher scores indicating better outcomes. Positive scores indicate improvements in KOOS_4_, and negative scores indicate a decline in KOOS_4_. PNE indicates pain neuroscience education.

The responder analysis showed that 8 of 24 participants (33.3%) in the neuromuscular exercise and PNE group and 8 of 22 participants (36.4%) in the PNE-alone group (16 of 46 total participants [34.8%]) experienced clinically important improvements (10 points) from baseline to the 12-month follow-up for the primary outcome KOOS_4_. Individual changes in KOOS_4_ from baseline to 12 months are shown in Figure 3. There was no difference in the proportion of responders between the groups (relative risk, 1.09; 95% CI, 0.49-2.41; *P* = .83).

There were no significant between-group differences in change in the 5 KOOS subscales of pain, symptoms, activity of daily living, sport and recreation, and knee-related quality of life; the global perceived effect; time to complete the 40-m fast-paced walk test and the stair-climb test; or numbers of repetitions in the 30-second chair-stand test (Table 2). Nor was there a significant between-group difference for use of pain medication (relative risk, 1.02; 95% CI, 0.73-1.43; *P* = .92) (eTable 2 in Supplement 2). Both groups experienced significant within-group improvements in all outcomes except use of pain medication, in which neither group showed an improvement; the KOOS subscale sport and recreation, in which the neuromuscular exercise and PNE group showed no improvement; and the 40-m fast-paced walk test, in which the PNE-alone group showed no improvement.

No serious adverse events were registered in either of the intervention groups during the trial. For the neuromuscular exercise and PNE group, 5 nonserious adverse events were registered during the trial: 4 participants experienced increased pain intensity, and 1 participant experienced swelling in the index knee following a neuromuscular exercise session, which subsided after a few days and did influence the next neuromuscular exercise session. No nonserious adverse events were registered in the PNE-alone group. No participants in either group reported that they had received other treatments during the trial period. The per-protocol analysis revealed no differences in changes from baseline to 12 months for neither the primary nor the secondary outcomes (eTables 3 and 4 in Supplement 2).

## Discussion

To our knowledge, the NEPNEP trial is the first randomized clinical trial evaluating exercise and education for patients with chronic pain after TKA. Our results revealed that neuromuscular exercise and PNE were not superior to PNE alone for the primary outcome KOOS_4_ in patients with chronic pain after TKA or for any of the secondary outcomes. Consequently, the results did not support the hypothesis that neuromuscular exercises and PNE would lead to greater improvements in pain and function than would PNE alone. We observed clinically important improvements in approximately one-third (34.7%) of the participants with chronic pain after TKA, regardless of treatment allocation.

Studies evaluating the effect of treatments introduced in the early postoperative period^32,33,34,35,36,37^ have not considered that patients who undergo TKA often experience spontaneous improvements in pain between 3 and 9 months after surgery.^38^ Hence, the observed treatment effects could have been influenced by the natural course of improvement after TKA and are therefore not generalizable to patients with chronic pain more than 1 year after TKA. Our findings contribute insight into the treatment of the patients who do not experience spontaneous improvements postoperatively and still experience chronic pain for at least 1 year after their TKA surgery.

Qualitative research has shown that patients with chronic pain after TKA feel abandoned by the health care system and the lack of treatment options. Therefore, patients experience their pain as something they are stuck with and that nothing more can be done.^10^ Our results challenge that perception. Given that both intervention groups experienced similar outcomes, the introduction of PNE as treatment could be of particular importance. By providing PNE, patients might realize the factors they can influence themselves, which could lead to improved self-management.

As illustrated in Figure 2, the neuromuscular exercise and PNE group exhibited an improvement in KOOS_4_ immediately after the 3-month supervised exercise therapy program. While the neuromuscular exercise and PNE-alone group largely maintained their improvements until the 12-month follow-up, the PNE group gradually improved from baseline to 12 months. This could indicate that exercising is effective when performed with effects diminishing over time, similarly to findings within hip and knee osteoarthritis.^39,40^ Therefore, it would be valuable to investigate whether a longer period of exercise therapy or booster sessions could provide sustained improvements.

The KOOS was chosen as the primary outcome, as it is imperative to consider the patient perspective when evaluating treatment effect.^41,42^ The psychometric properties of KOOS have been scrutinized, with some findings indicating the need for further validation^42^ and other findings consolidating its validity and reliability.^23,41^ However, the KOOS remains a frequently used patient-reported outcome measure for patients undergoing TKA.^17,43,44^

As illustrated in Figure 3, participants from both groups experienced large improvements in KOOS_4_, highlighting that some participants benefited substantially from neuromuscular exercise and PNE or PNE alone. On the contrary, other participants in both groups experienced little improvement or even a worsening in KOOS_4_. This supports the need for individualized approaches when seeking the best possible treatment. Future research should investigate which patient characteristics indicate a favorable response to exercises and PNE and who might not benefit from either.^45^

The mean body mass index in our population was greater than 33, most participants had at least 1 comorbidity, and there was a group-average score in the Hospital Anxiety and Depression Scale^31^ that indicated clinical depression. These factors have previously been associated with chronic pain after TKA^45^ and emphasize the complexity of the studied population. Given the multiple factors influencing chronic pain and the characteristics of the population, a biopsychosocial and multimodal treatment approach should be considered for patients with chronic pain after TKA.^6,10^

### Limitations

This trial has some limitations. The study was affected by the COVID-19 pandemic and failed in recruiting the target sample size. However, when taking the small between-group differences into consideration, it seems unlikely that a fully powered study would change the conclusion of no between-group differences. Moreover, considering that the study did not include a no-treatment control group, the true effects of neuromuscular exercises and/or PNE could not be determined. Therefore, the findings could represent fluctuations in pain intensity over time. Long-term follow-up studies have observed that some patients experience pain fluctuations after TKA, whereas other patients’ chronic pain remains stable over time.^38^

## Conclusions

The results of this randomized clinical trial suggest that neuromuscular exercises and PNE were not superior to PNE alone for the primary outcome on pain, symptoms, function, and knee-related quality of life or any of the secondary outcomes in participants with chronic pain after TKA. The study demonstrated clinically relevant improvements in approximately one-third of the participants, regardless of intervention group. This finding challenges the perception that nothing can be done to relieve pain in patients with chronic pain after TKA. Therefore, the results could have important implications for the future management of patients with chronic pain after TKA. Despite the contributions of this study, an evidence gap for the treatment and management of patients with chronic pain after TKA remains and should be further addressed in future research.

## Notes

Supplement 1.
Trial Protocol and Statistical Analysis Plan

Supplement 2. **eAppendix 1.** CONSORT Checklist for Randomized Trials **eAppendix 2.** TIDieR Checklist for Information to Include When Describing an Intervention **eAppendix 3.** CERT Checklist for What to Include When Reporting Exercise Programs **eMethods 1.** Pain Neuroscience Education Session 1 **eMethods 2.** Pain Neuroscience Education Session 2 **eTable 1.** Patient Baseline Characteristics for Those Attending the 12-Month Follow-up Assessment and Those who Did Not Attend **eTable 2.** Intention-to-Treat Analysis for Risk Ratios for Usage of Pain Medication From Baseline to 12 Months **eTable 3.** Per-Protocol Analysis for the Primary and Secondary Outcomes for Change From Baseline to 12 Months **eTable 4.** Per-Protocol Analysis for the Changes in Usage of Pain Medication From Baseline to 12 Months

Supplement 3.
Data Sharing Statement
