# Supplementary material for: Exercise and Pain Neuroscience Education for Patients With Chronic Pain After Total Knee Arthroplasty: A Randomized Clinical Trial
Source: JAMA Netw Open. 2024 May 24;7(5):e2412179. doi: 10.1001/jamanetworkopen.2024.12179 (PMC11127128; doi:10.1001/jamanetworkopen.2024.12179)
Supplement: Supplement 1. — Trial Protocol and Statistical Analysis Plan [file jamanetwopen-e2412179-s001.pdf]

Research protocol

Project title

Neuromuscular exercise and pain neuroscience education compared to pain neuroscience education alone in patients with chronic pain after primary total knee arthroplasty: A randomized controlled trial

Principal investigator and responsible for the study

Jesper Bie Larsen, Ph.d., physiotherapist.
Sport Sciences og Forskningsenheden SMI
Department of Health Science and Technology
Aalborg University
Niels Jernes Vej 12, Room A5:205
9220 Aalborg East
E-mail: jbl@hst.aau.dk
Phone: +45 28 26 84 59

Clinical responsible

Mogens Laursen, MD, Ph.d.
Orthopedic Surgery Research Unit
Aalborg University Hospital
Hobrovej 18-22,
9000 Aalborg
Phone: 9766 2502
E-mail: mola@rn.dk

Projektgroup

- Lars Arendt-Nielsen, dr.med., Ph.d., Professor, SMI, Aalborg Universitet
- Pascal Madeleine, dr.scient., Ph.d., Professor, Sports Sciences, Aalborg Universitet
- Jesper Bie Larsen, Ph.d., PT., Sports Sciences & SMI, Aalborg Universitet
- Ole Simonsen, Overlæge, dr. Med, Ortopædkirurgisk Afdeling, Klinik Hoved-Orto
- Mogens Laursen, Overlæge, Ph.d., Ortopædkirurgisk Afdeling, Klinik Hoved-Orto
- Søren Thorgaard Skou, Ph.d., PT., Professor, Institut for Idræt og Biomekanik, Muskuloskeletal Funktion og Fysioterapi (FoF) og Syddansk Universitet og Næstved, Slagelse og Ringsted Sygehuse.

Experimental Protocol

Background

Multiple studies have examined osteoarthritis (OA), its current treatment concepts, and the effects of these. OA is considered the most frequent cause of disability and pain in the elderly population, and the knee joint is one of the joints most commonly affected[1,2]. Since end-stage OA is often treated with knee replacement, the number of total knee arthroplasties (TKA) performed has increased over the past years, and the increase is expected to continue due to a growing elderly population, an increased prevalence of obesity and a sedentary lifestyle among both elderly and young people[3]. Primary TKA is considered an effective treatment for pain relief and improved function[4,5]. However, several studies have reported less favorable outcomes after primary TKA, and two systematic reviews found chronic pain rates after primary TKA at 12 months post-operative in 13-17% of the patients and chronic pain rates at 2-7 years post-operative varying between 8-27%[6,7]. As for pain intensity, a study by Wylde et al. (2011) observed that 15% of their primary TKA cohort, consisting of 632 patients, 3-4 years post-operatively reported their pain intensity as severe-to-extreme (defined as WOMAC Pain score <50).

In recent years, much interest has been given to the underlying mechanisms of pain in OA. Chronic pain in OA patients is believed to occur as a result of local pathological processes in the joint, genetic and metabolic factors and neuronal changes at several levels, including peripheral or central sensitization, reduced descending inhibition and atrophy of cortical areas[8]. The International Association for the Study of Pain defines sensitization as "*increased responsiveness of nociceptive neurons to their normal input, and/or recruitment of a response to normally subthreshold inputs", and sensitization has emerged as* an important mechanism in the development of chronic pain. Several studies have observed evidence of sensitization in patients with knee OA[9-12] and a study by Wright et al. (2015) investigated whether sensitization persists after TKA and found abnormal Quantitative Sensory Testing (QST) – indicating sensitization - in patients, with persistent pain after TKA, 12-36 months post-operatively[13].

Regarding muscle strength and function, a vast amount of literature has examined the outcomes after primary TKA. To sum up the evidence, a systematic review and meta-analysis found that quadriceps muscle strength was significantly lower compared with age- and sex-matched controls at all included time-points (4-6 months, 1-3 years and >3 years post-operatively)[14]. Regarding functional capacity after TKA, a systematic review reported that 20% of patients felt that surgery had not been successful enough for them to resume regular functional activities. Forty percent of patients used a walking aid during walking after TKA and in general, patients reported difficulties walking, stair climbing, shopping, dressing and getting in and out of bed[6].

Several rehabilitation regimes for pain relief and improving function in the post-operative period following TKA have been proposed, examining a variety of exercises, pre-operative or post-operative rehabilitation and exercise intensity following surgery[15-22]. Conflicting results exist and no conclusive evidence regarding exercise type, initiation, frequency,

duration or intensity has been established, making treatment recommendations difficult. Therefore, a lot of variation exists in rehabilitation protocols, which was recently highlighted in a survey study, revealing major differences in the rehabilitation offered to patients after primary TKA in different hospitals in Denmark[23]. The above-mentioned studies were all conducted in patients with a “normal” progression post-operatively whereas the evidence for patients with chronic pain and functional impairment after TKA is scarce. To underline this, two studies recently conducted systematic reviews with the purpose of gathering evidence-based knowledge regarding the management and treatment of chronic pain after surgery, including after TKA[24,25]. The studies found no randomized controlled trials evaluating physical treatment to patients with chronic pain after TKA, and both studies highlight the need for evidence-based knowledge of which treatment should be considered for this population. As of today, no standardized treatment regime exists for patients with chronic pain after TKA. Case studies of treatment effects exist, such as Bhave et al. (2005), who observed improved function after an intensive rehabilitation regime provided to patients with substantial dysfunction[5]. This study did not include any pain measurements or core outcome measures as proposed by The Osteoarthritis Research Society International (OARSI), which makes it difficult to generalize to a group of patient with chronic pain after TKA.

A neuromuscular training program developed by Ageberg et al. (2010) has been shown to be feasible and effective in treating pain and impaired function in patients with knee OA[26]. The same neuromuscular training program was used in a recent randomized controlled trial investigating the additional effect from primary TKA in addition to conservative treatment[27]. The study revealed that primary TKA followed by a 12-week rehabilitation regime was more effective in relieving pain and improving function compared with rehabilitation alone. However, the patients that received rehabilitation alone experienced clinically relevant improvement and had less serious adverse events than the group receiving primary TKA and rehabilitation. The neuromuscular rehabilitation program emphasized re-learning and re-establishing of normal motor program strategies by including sensorimotor control training[28]. This could be relevant for patients with chronic pain as altered movements and changes in muscle activation have been observed, leading to a decrease in physical performance, and this may be a part of the maintenance of pain[28,29]. Hence, the neuromuscular rehabilitation program used by Skou et al. (2015) and previously developed by Ageberg et al. (2010) is an effective rehabilitation program in patients with knee OA and after primary TKA. It is unknown if similar results can be observed in a population of patients with chronic pain and impaired function in the late stages after primary TKA.

Preoperative education has been used for many years in an attempt to decrease postoperative pain and anxiety and to improve recovery after different surgical procedures. A vast variety of content in the preoperative education has been proposed, including information of surgical procedures, post-operative pain and pain education, anatomy and function of normal joints and patho-anatomy of arthritic joints, specific movements to avoid and activities of daily living[30,31]. Preoperative education compared with usual care has shown conflicting results with suggestions that it might reduce anxiety and reduce the length of hospitalization[30,31]. Others have reported no positive effect on post-operative pain

or function, which was the conclusion in a Cochrane review by McDonald et al. (2014)[32]. Recently, a shift in education from the abovementioned biomedical model to a biopsychosocial model has been proposed as education for chronic pain patients[33,34]. Since sensitization is a frequent phenomenon in chronic pain patients, a new approach to education is needed; one that targets pain neuroscience instead of focusing on the nociceptive pain[33]. Pain Neuroscience Education (PNE), an education program consisting of knowledge of neuroscience and terms as sensitization, hyperalgesia and allodynia, has been investigated in recent years. Results have been conflicting without establishing strong evidence-based suggestions for PNE[33-35]. PNE has only been examined as a pre-operative intervention and therefore it is unknown whether post-operative education given to chronic pain patients after TKA will have any positive effect on pain, anxiety, quality of life or function.

Strategy for Literature Search

The basis of the study has been found among peer-reviewed articles, publicly available material and Aalborg University's own research. 962 articles were found and 52 references used (please see the section List of References) for this study forming the basis for the research within chronic pain in patients after receiving primary TKA in this study.

The literature has been found by reviewing of the search engines Pubmed, Cochrane Library, Embase and CINAHL. The following search words were used in combinations for review of the literature: Knee osteoarthritis/ total knee arthroplasty/ total knee replacement/ pain/ chronic/ persistent/ sensitization/ quantitative sensory testing/ function/ functional capacity/ neuromuscular exercises/ pain neuroscience education.

Only studies with interest within human research have been used.

Purpose

The main purpose of the study is to evaluate whether a 12-week neuromuscular rehabilitation program combined with PNE provides greater pain relief and improvement in function and quality of life than PNE alone in a population of patients with chronic pain after primary TKA.

Hypothesis: Rehabilitation involving neuromuscular training and PNE will provide pain relief, improved function and higher quality of life.

The secondary purpose is to evaluate the effects of neuromuscular rehabilitation and PNE in terms of sensitization in those patients with sensitization after primary TKA.

Hypothesis: Rehabilitation involving neuromuscular training and PNE will decrease sensitization.

Subjects

For this study, we will recruit 120 patients with chronic pain after primary TKA. The patients will be included by the staff at the orthopedic surgical department at Aalborg University Hospital. In order to recruit patients, the clinically responsible doctor will screen the medical charts of patients who have had primary TKA. Eligible patients will be contacted by either letter or email (see the letter in "Opslag_v1_28052018). Further, potential subjects will be contacted by phone or by personal approach by healthcare personnel when attending the orthopedic department at Aalborg University Hospital.

The following inclusion and exclusion criteria will be used:

Inclusion

- Male or female aged 40-80 years
- Body Mass Index (BMI) between 19-40
- Subjects with primary TKA due to osteoarthritis ≥ 12 months post-operatively
- For the index knee, duration of knee pain > 6 months
- For the index knee, the average daily pain score ≥ 4 (moderate-to-severe pain) over the last week prior to visit on a numeric rating scale (NRS) scale

Exclusion

- Specific reasons for chronic pain, such as loosening of implant, which requires revision surgery
- Secondary causes of arthritis to the knee
- Surgery (including arthroscopy) of the index knee within 3 months prior to visit
- Injury to the index knee within 12 months prior to visit
- Recent history of acute pain affecting the lower limb and/or trunk
- Participation in other pain trials two weeks prior to this study
- Pregnancy
- Drug and alcohol abuse
- Previous neurologic illnesses or primary pain area other than knee, e.g. low back, upper extremity pain or rheumatoid arthritis
- Lack of ability to adhere to protocol

Design and Methods

The design is a randomized controlled trial. Patients will be randomly assigned in a 1:1 ratio, using a computer-generated randomization list, to undergo either 12-weeks of rehabilitation combined with PNE or PNE alone. All assessments will be conducted at Fysio- og Ergoterapiafdelingen, Afsnit A, Aalborg Universitetshospital, Hobrovej 18-22, 9000 Aalborg. Interventions will be conducted at one of the following locations, based on geography and the patient's preference:

Fysio- og Ergoterapiafdelingen, Klinik Hoved-Orto, Afsnit A, Aalborg Universitetshospital, Hobrovej 18-22, 9000 Aalborg.
Fysio- og Ergoterapiafdelingen, Klinik Hoved-Orto, Aalborg Universitetshospital, Højgårdsvej 11, 9640 Farsø
Fysio- og Ergoterapiafdelingen, Klinik Hoved-Orto, Afsnit D, Aalborg Universitetshospital, Højtoftevej 2, 7700 Thisted

The 12-week rehabilitation program consists of a neuromuscular exercise program, which has previously been shown feasible in patients with moderate-to-severe knee OA and in patients after TKA[26,27]. The exercise program will be conducted in a 1-hour, group-based session twice a week for 12 weeks (24 sessions in all, 1 hour per session). Specially trained physiotherapists will instruct and supervise the patients during the neuromuscular exercises. The aim of the neuromuscular exercise program is to restore normal movement, improve sensorimotor control, re-establishing normal motor program strategies and muscle activation[26,36]. Correct posture and alignment will be emphasized during exercises and will be monitored individually to ensure good quality of the movements. Pain levels during and after training will be monitored and patients will be informed that pain the day after training should be “pain as usual”, otherwise the intensity and volume of training will be reduced[26]. Due to the chronicity of the patients’ pain, a time-contingent approach to the exercises will be preferred over a symptom-contingent method[37]. The content of the exercise program can be seen in appendix.

The PNE consists of two group-based information meetings; one at the start of the study period and one 6 weeks after initiation of the study. Both sessions will take 1-hour and will be conducted by specially trained physiotherapists. The aim of the PNE is to increase the pain neuroscience knowledge of the patients leading to a better understanding of their chronic pain and thereby engaging the patients in the treatment of their chronic pain and impaired function[38]. During the sessions, it will be possible to ask questions and share experiences within the patient group.

Follow-up assessments
Baseline measurements will be assessed before initiation of intervention and follow-up measurements will be performed at 3, 6, 12 and 24 months after initiation of the intervention. Each assessment is expected to take approximately two hours for each participant. The same specially trained assessor, who will be blinded, regarding which treatment group the patients belong to, will do all assessments.

Outcomes
The following assessments will be performed:

Questionnaires:
- Knee Osteoarthritis Outcome Score (KOOS)
- PainDETECT questionnaire
- Hospital Anxiety and Depression Scale (HADS)
- Fear-avoidance Beliefs Questionnaire – Physical Activity (FABQ-PA)
- Global Perceived Effect (GPE)

- Pain Catastrophizing Scale

Pain measurements:
- Maximal pain intensity during rest (day and night), stair climbing, and walking on a Numeric Rating Scale (NRS)
- Pain drawings of pain distribution
- Bed-side-test measurements to examine sensitization
- Use of painkillers

Physical performance outcomes:
- 40-meter fast-paced walk test
- Stair climb test
- 30sec. chair test
- Leg extension power
- Muscle strength of knee extensors (quadriceps), knee flexors (hamstrings) and hip abductors (gluteus medius)

Adverse events
- Serious and non-serious adverse events

Other treatments received
- Registration of other treatments received

Compliance to interventions
- Number of attended sessions

Primary outcome
Knee Osteoarthritis Outcome Score (KOOS)
KOOS is a subjective questionnaire covering five subscales of pain, symptoms, activities of daily living, sports/recreation and quality of life. Each KOOS subscale consists of multiple items, scored on a 5-point Likert scale; The KOOS ranges from 0 (worst) to 100 (best)[39]. KOOS is a patient self-reported outcome measure, which is found valid and reliable during short-term and long-term follow-up in patients with TKA[40]. In the present study, the change from baseline to 12-month follow-up of the mean score of 4 subscales evaluating pain, functions, activities of daily living and quality of life (KOOS^4) will be used as primary outcomes, which is in accordance a previous study within the field of osteoarthritis[27]. A change of more than 10 points will be considered clinically relevant[41].

Secondary outcomes

Questionnaires
Knee Osteoarthritis Outcome Score (KOOS)
The scores on all 5 subscales, including the sport-recreation subscale, of KOOS are included in order to assist the clinical interpretation of the primary outcome[42].

PainDETECT Questionnaire
The PainDETECT Questionnaire has been developed to assess the neuropathic components of a given pain disease[43]. The PainDETECT is a validated, easy to use, screening tool that predicts the likelihood of a neuropathic pain component in chronic pain disorders. The questionnaire is comprised of 3 major components: graduation of pain, pain course pattern and radiating pain. Seven questions evaluate the graduation of pain. Each question is scored by the subject using a 0 to 5 score with 0 = never, 1 = hardly notice, 2 = slightly, 3 = moderately, 4 = strongly and 5 = very strongly. One question evaluates the pain course pattern. The subjects select one of four pictures to indicate the pattern of pain best describing their course of pain. Each picture is associated with a unique score of 0, -1, or +1 (2 different pictures have this score). One question evaluates radiating pain with a yes (score of +2) or no (score of 0) response option. The PainDETECT is calculated by addition of the subject's responses to all questions. Thus, the maximum possible score is 38, and the minimum possible score is -1. Only integral values are possible. Total PainDETECT scores of ≤ 12 indicate that a neuropathic pain component is unlikely (< 15%). Total PainDETECT scores of ≥ 19 indicate that a neuropathic pain component is likely (>90%). Scores of 13 to 18 are uncertain.

Hospital Anxiety and Depression Scale (HADS)
The HADS is a self-reported questionnaire with 14 items, which measures the patient level of anxiety and depression. The HADS consists of two subscales, measuring anxiety (HADS-A) and depression (HADS-D). The score in each item ranges from 0-3, giving separate scoring ranges from 0-21 in each subscale. A score is considered normal within the range of 0-7, borderline abnormal within the range of 8-10 and abnormal anxiety and/or depression within the range of 11-21[44]. The HADS has been found a reliable and valid tool for measuring anxiety and depression[45].

Fear-avoidance Beliefs Questionnaire – Physical Activity (FABQ-PA)
The FABQ is a 16-item questionnaire originally developed for patients with low back pain. The questionnaire consists of two subscales regarding work and physical activity. In the present study the subscale regarding physical activity will be used. FABQ-PA is a 4-item questionnaire in which each item provides a seven-category Likert-scale with scoring alternatives from "completely disagree" to "completely agree". The scores range from "0" with "completely disagree" to "6" with "completely agree" and sums up to a total score between 0-24. A high score indicates a high degree of fear-avoidance beliefs[46]. For the present study, only the subscale FABQ-PA will be used to address fear-avoidance in relation to physical activity in patients with chronic pain after TKA and therefore the word "back" has been replaced with "knee" and the examples of physical activity of "bending, lifting, walking and driving" has been replaced with "running, walking, kneeling and driving". This approach has previously been used in other studies within the field of osteoarthritis[47-49].

Global Perceived Effect (GPE)
GPE will be assessed using the question: "How are your knee problems now compared with before you entered this study"? The question will be answered on a seven-point Likert

scale ranging from 'Improved, an important improvement' to 'Worse, an important worsening'. The use of GPE scales has been found reliable in a previous study[50].

Pain Catastrophizing Scale
The PCS is a 13-item questionnaire developed to explore how catastrophizing affects pain experiences. The questionnaire includes the subscales rumination, magnification, and helplessness. Scores is rated on a 5-point scale of frequency with the 0 being "not at all" and 4 being "all the time" and the score can range from 0 to 52 points[51]. It has been suggested that a threshold of 30 points reflects clinically relevant catastrophizing[52,53].

Pain measurements
Pain intensity NRS ratings
For the index knee, the average daily pain NRS intensity score over the last week prior to the visit will be assessed. Furthermore, the maximal pain intensity during rest (day and night), stair climbing, and walking will be assessed. "0" represents "no pain" and "10" represents "maximal pain". A change of minimum 2 points on the NRS will be considered clinically relevant[54].

Pain drawings
The patients will be asked to draw their habitual pain distribution on anatomical body maps (posterior and anterior views). Furthermore, the Knee Pain Map[55] will be used in order to assess whether the knee pain can be categorized as localized, regional or diffuse. The Knee Pain Map is an interviewer-administered assessment of knee pain patterns and location. The patients will point out on their own knee where the pain is perceived and the interviewer will record the pain location on an artist´s drawing of a knee and classify the pain as localized, regional or diffuse.

Bed-side-tests for sensitization
All bed-side tests will be performed in the area of the most affected knee, adjacent to the knee (10cm above the knee, ventral thigh), and extra-segmentally on the medial side of the forearm (muscle belly of flexor digitorum superficialis) unless stated otherwise.

<u>CMS Pinprick hyperalgesia</u>
A CMS (Chicago Medical Supply) nylon filament (0.7mm) will be applied perpendicularly to the skin (90° angle, slight bending of the hair). The subject is to rate the pain intensity of the pinprick on a NRS of 0-10 (0=no pain, 10=extreme pain).

<u>Temporal summation (Wind-up)</u>
The CMS nylon filament (0.7mm) will be applied perpendicularly to the skin (90 angle, slight bending of the hair) once and the subject is to rate the intensity of the sensation. After this, the CMS nylon filament will be applied 10 times in an area of 1 cm^2 with a frequency of 1/s and the subject is to rate the intensity of the last stimulus on a NRS of 0-10.

<u>Dynamic mechanical allodynia (DMA)</u>

The skin will be stroked with a cotton swab 4 times (twice from each direction of a cross with 90° angles). The length of each stroke will be 3-5cm. The subject is to rate the pain intensity of the cotton swab on a NRS of 0-10.

Deep somatic hyperalgesia
A ”bedside algometer (syringe)“ will be applied on the skin over the vastus medialis muscle on the affected side. The air in the syringe will be compressed with a constant speed (1ml per second) until the pressure becomes painful. The subject is to indicate immediately when the pressure becomes painful (threshold in ml).

Pressure Pain Threshold
A handheld algometer (Somedic, Hörby, Sweden) with a 1-cm2 probe (covered by a disposable latex sheath) is used to record the pressure pain threshold (PPT). The PPT is defined to the subject as ‘‘the point at which the pressure sensation just becomes painful.’’ The pressure is increased gradually at a rate of 30 kPa/s until the pain threshold is reached and the subject presses a stop button. The PPTs will be measured three times for each point in a random order and with an interval of minimum 20 seconds is between each PPT assessment. The average of the three recordings will be calculated for further analysis.

Descending pain control (CPM)
A descending pain control will be evoked by a 1.3 kg pressure clip applied to the ipsilateral earlobe or an ipsilateral fingernail.
The pain intensity will be assessed twice to a 6 kg 10 sec standardized pressure applicator applied to the contralateral (non-affected side) mid tibialis anterior muscle.
The pain ratings will be determined before and after 60 sec of the tonic earlobe or fingernail pain stimulation.
The subjects are to rate the evoked pain at the end of the 60 sec clip application on a Visual Analog Scale (VAS) on which one end of the scale represents “no pain” and the opposite end represents “maximal pain”.

Use of painkillers
Use of painkillers last week (yes/no), number of Paracetamols (1g), Ibruprofen (400 mg) and other NSAIDs last week.

Physical performance outcomes
The included physical performance measures, related to activity of daily living, are the recommended core set of outcome measures from OARSI[56].

40-meter fast-paced walk test
This test evaluates the walking speed and the ability to walk a short distance. The participants are asked to walk as quickly and as safely as possible, without running, along a 10 m walkway and then turn around a cone, return, and then repeat for a total distance of 40 m. Regular walking aid is allowed. If safety is of concern, the tester will follow slightly behind and off to one side of the participant but not as to pace or impede the participant. The amount of time it takes to complete the 40m is the outcome score[56].

30-second chair stand test
The test is a test of sit-to-stand activity and thereby a test of lower body strength and dynamic balance. From the sitting position, the participant stands up completely so hips and knees are fully extended, then sits completely back down so that the bottom fully touches the seat. This is repeated for 30 seconds and the number of repetitions is the outcome score. If the person cannot stand even once, then the hands are to be placed on the legs or the regular mobility aid is to be used[56].

Stair climb test
The stair climb test is a test of ascending and descending stair activity and thereby a test of lower body strength and balance. The subject is to ascend and descend a staircase with approximately 9 stairs once. The subject is asked to ascend and descend the stairs as quickly and as safely as possible. Use of a handrail and walking aid is permitted, if needed. The amount of time it takes to complete the ascending and descending of the stairs is the outcome score[56].

Leg extension power
Leg extension power expressed as the product of force and velocity in a single-leg simultaneous hip and knee extension will be measured. The participant will be seated with their arms crossed, the operated leg placed on a footplate, and the other foot resting on the floor. They will be asked to push the pedal down as hard and fast as possible. The force will be recorded for each push (30 seconds rest between trials) until they reach a plateau defined as two successive measurements below the highest measurement. A minimum of six trials to minimize the learning effect and a maximum of 12 trials to minimize fatigue, will be obtained and the highest measurement in watt will be used as the data point[57].

Muscle strength of knee extensors, flexors and hip abductors
Maximal voluntary isometric contractions from the knee extensors and flexors and the hip abductors will be measured bilaterally with a hand-held dynamometer (Lafayette Manual Muscle Tester). For the knee extensors, the participant will be seated (90° of hips and knees flexion) and the dynamometer will be placed on the anterior aspect of the tibia, proximal to the ankle joint. During the test, the participant will extend the knee against the dynamometer. For the knee flexors, the participant will be seated as explained above and the dynamometer will be placed on the posterior aspect of tibia, proximal to the ankle joint. During the test, the participant will flex the knee against the dynamometer. For the hip abductors, the participant will be in supine position with hips and knee extended and will be asked to abduct his/her test-leg against the dynamometer, placed proximal to the lateral malleol[58,59]. For all three tests, the participant will be asked to exert a maximum effort of 5 seconds against the hand-held dynamometer as fast as possible. A submaximal practice trial will be performed to assure that the participant performs the correct movement. Three trials of each test will be performed and the mean score in Newton-meters/kg of these is the outcome score[58]. Further, the rate of force development will also be measured[60].

Adverse events
Serious and non-serious events that may occur during the study period will be identified in different ways: by self-reporting by the participants and by observation from the physiotherapists. Adverse events will be categorized as occurring in the index knee or other sites than the index knee and serious events will be identified according to the definitions from the U.S. Food and Drug Administration[61]. Non-serious adverse events comprise of all other events occurring.

Other treatments received
Other sort of treatments received in the intervention period will be identified by self-reporting. Other treatment is defined as treatments that the participant has initiated because of the index knee (e.g., acupuncture, manual therapy). Duration and frequency will be registered.

Compliance to interventions
Compliance will be registered for both groups. In the neuromuscular exercises and PNE group the number of attended exercise sessions and PNE sessions will be recorded (i.e. number of sessions out of 24 exercises sessions and two PNE sessions). In the PNE group the number of PNE sessions will be recorded (i.e. number of sessions out of two possible sessions). Poor compliance will be defined as participating in less than 75% of the exercise sessions.

Risks, Side Effects and Disadvantages

The pain assessment methods, the physical performance tests as well as the neuromuscular exercises used are all well-proven and have all been performed in in similar studies. There are no reports from our or other institutions on long-term side effects of the methods. The applied assessments can be interrupted at any time. The subjects will be under observation during the trial.

The subjects will experience a weak short-lasting pain during the pain assessment. Specifically, the pressure stimulation will cause a pressing or clamping pain. However, the pain disappears immediately after the termination of the stimulations and does not cause any side effects.

During the physical performance exercises and testing, the subjects may experience pain when performing the chair stand test, the walking test, the stair climb test and the muscle strength test as well as during the exercises in general. The exercises and tests are commonly used in clinical settings and are therefore considered feasible and safe. Any discomfort from these tests is expected to diminish shortly after the test has ended.

The exercises may induce muscle soreness, which will disappear a day or two after the training session. Pain after exercises should not exceed “normal” pain intensity; otherwise, exercise intensity will be adjusted. A physiotherapist supervises all training sessions and provides individualization of the exercises where required.

Statistics

Between-groups comparisons of change from baseline to the primary endpoint, the 12-month follow-up, will be conducted for the primary and secondary outcomes. Statistical tests will be dependent on data distribution. We expect data to be normally distributed and therefore for continuous outcomes we will be using a repeated measures mixed model with patients as random effect and visit (baseline, 3, 6 and 12 months) and treatment arm (neuromuscular exercises and PNE or PNE alone) as fixed effects, and with adjustment for baseline imbalance and the randomization stratification factor (gender). No imputation will take place. P-values and 95% CI will be presented. A CI excluding 10 points or more in the KOOS$_4$ score will be interpreted as a lack of a clinical meaningful difference. Frequency of adverse events will be compared between groups at the 12 months follow-up using a Poisson regression model with a robust error variance. Categorical outcomes will be analyzed using X^2 test, Fisher's exact test or Mann-Whitney U test as appropriate. Validation of normal distribution will be done by assessing the data frequency in histograms and tests for normality (Shapiro-Wilk).

All randomized patients will be included in the intention to treat analysis and in the safety analysis. A per protocol will be performed for the primary outcome excluding patients who have poor compliance with the exercise (participating in less than 75% of the exercise sessions) and patients who undergo revision knee replacement surgery.

Sample size calculations

For KOOS^4 and KOOS scores, a minimal clinically important difference of 10 is estimated, and commonly used[39]. A sample size calculation was conducted in order to estimate the sample size required to give the study a power of 90% to detect a minimum improvement of 10-point on the KOOS^4 in the neuromuscular exercise and PNE group compared with the PNE group (with a standard deviation of 15)[27,41]. A two-sided significance level at 0.05 was applied and results revealed that 49 participants are required in both groups. To account for possible missing data and a loss to follow-up of 20%, a total of 60 participants in each group will be enrolled.

Ethical Considerations

The study complies with the Helsinki Declaration and is submitted to the North Denmark Region Committee on Health Research Ethics for approval. The methods used have been tested and performed in several studies, both in Denmark and abroad, and no long-term side effects have been reported. As described above, the risks associated with the experiments are considered minimal and we do not expect other risks to emerge during the project.

Approximately 13-20% of patients who have had a primary TKA suffer from chronic pain and impaired function leading to disability and lack of quality of life[6,7]. Recent evidence suggests that central sensitization can be one of the reasons behind the chronic pain[9-12,62]. Therefore, development of treatments targeting the nervous system as well as the physical parameters is essential to provide the optimal treatment.

The current study will introduce mild to moderate but, short-lasting, pain to the subjects, but the outcome from this study can lead to a clinically useful treatment for patients with chronic pain after primary TKA. The subjects will potentially benefit from decreasing pain intensity and increasing muscle strength as a result of the exercises. However, it cannot be guaranteed that all subjects will benefit from the exercises and the duration of the benefits cannot be guaranteed. Further, the subjects cannot continue the exercise in these settings after the termination of the project. Finally, the PNE will potentially give the subjects a better understanding of their chronic pain and thereby, possibly engage them in any future treatment of their chronic pain and impaired function.

Insurance

The subjects are covered by the Danish Patient Compensation Association (Patient-erstatningen).

Placebo/Control Treatment

The control treatment consists of the PNE program.

Personal Data

Data will be stored after termination of the project. These data can only be used for the interpretation of this project and will therefore not be of interest to third party.

Data are stored in accordance with the stipulations in The Danish Personal Data Protection Act (Persondataloven) and other relevant Danish legislation.

The project is reported to The Danish Data Protection Agency through the Aalborg University umbrella agreement

Information from Medical Charts

The clinically responsible doctor will retrieve information about the surgical procedures and previous pain intensity from patient records prior to inclusion of the subjects. This is necessary to be able to identify and approach eligible patients. If the subject consents to participation, relevant information about the osteoarthritis and the TKA surgery will be passed on to the researcher responsible for the principal investigator (forsøgsansvarlig) in order for him to conduct quality assurance of the project.

Project Economy

The project has been initiated by Jesper Bie Larsen, Ph.D. and physiotherapist, Sports Sciences and SMI, Department of Health Science and Technology, Aalborg University.

The project is financed with DKK 2 mio by the Department of Health Science and Technology, Aalborg University, DKK 235.000 by the Danish Rheumatism Association and DKK 700.000 by the Svend Andersen Foundation.

None of the researchers involved has financial interests in the study.

Compensation to Subjects

The subjects will not receive compensation for their participation, but they will be able to have their documented travel expenses reimbursed, according to current rates.

Publishing of Results

All results of the project will be published regardless of the outcome of the project.

Time Schedule

The experimental study will start after approval in September 2018 and will be completed in December 2025.

Guidelines for Oral Information and Informed Consent

Summoning Potential Subjects

The following should be stated when potential subjects, address the contact person on the basis of the letter/email received (see text in “Opslag_v1_28052018”), and when healthcare personnel contact potential subjects by phone or by personal approach in connection with their attendance at the orthopaedic clinic:

- That it is a request for participation in a scientific research project
- The purpose of the project
- That participation is voluntary and that the subject can withdraw from the project at any time without consequences
- That the potential subject has time to consider his/her participation before giving consent to participation in the project and that the potential subject is welcome to bring a family member or a friend to the information meeting. The potential volunteer will receive the leaflet “The Rights of a Trial Subject in a Health Scientific Research Project”/ "Forsøgspersonens rettigheder i et sundhedsvidenskabeligt forskningsprojekt" which includes information on confidentiality, right of access to documents and right to complain.
- That the material “Information for Participants”/”Deltagerinformation” will be forwarded by mail/e-mail to the potential subject in order for him/her to know more about the project before the information meeting.
- Finally, time for the information meeting is arranged

The Information Meeting

The information meeting is held in a quiet room where it is possible to have an uninterrupted conversation. Coffee/tea/soft drink may be served. The information meeting is held by the person responsible for the project or a senior researcher who has been authorized to do the information.

The meeting is to include the following information/questions:

- Participation is voluntary and the subject can withdraw from the project at any time without consequences
- The subject has time to consider his/her participation before giving consent to participation in the project, and the subject is welcome to bring a family member or a friend to the information meeting.
- The subject is asked whether he/she wants a family member/friend to be present at the meeting.
- The purpose of the experiment is presented, and it is explained how the experiment is performed. The "Information for Participants"/"Deltagerinformation", which has been sent to the potential subject in advance, is the starting point for the information meeting.
- The subject is asked if he/she is healthy or whether he/she has an infectious disease.
- The subject is asked whether he/she is a Danish citizen. If the answer is no, he/she is asked if he/she has a valid work permit.
- The leaflet "The Rights of a Trial Subject in a Health Scientific Research Project"/ "Forsøgspersonens rettigheder i et sundhedsvidenskabeligt forskningsprojekt" is handed over. It is explained that it includes information on confidentiality, right of access to documents and right to complain.
- The subject is asked whether he/she has read "Information for Participants"/ "Deltagerinformation". If this is not the case, we will ask the subject to read it.
- When it has been ensured that the subject has read the "Information for Participants"/"Deltagerinformation", he/she is asked whether he/she has questions about the experiment.
- After this a demonstration is given in the lab; measuring equipment and its use is presented to the subject.
- It is underlined that participation is voluntary, and that the subject has time to consider his/her participation (please note that The National Committee on Health Research Ethics recommends 24 hours of deliberation time)
- Again it is underlined that participation is voluntary and that the subject can withdraw his/her consent at any time without consequences.
- The subject is informed that if he/she does not need time to consider the participation, the consent can be given at the information meeting.
- Time/place for the experiment is agreed.

- Finally, information about the contact person of the experiment is given (it is shown to the subject that the name and contact details appear from the "Information for Participants"/"Deltagerinformation") and it is informed that this person can be contacted at any time if further questions should arise.

List of References

1. Peat G, McCarney R, Croft P. Knee pain and osteoarthritis in older adults: A review of community burden and current use of primary health care. *Ann Rheum Dis*. 2001;60(2):91-97.

2. Dieppe PA, Lohmander LS. Pathogenesis and management of pain in osteoarthritis. *Lancet*. 2005;365(9463):965-973.

3. Davidson D, de Steiger R, Graves S, Tomkins A et al. Australian orthopaedic association national joint replacement registry. annual report. adelaide:AOA;2010. . 2010.

4. Carr AJ, Robertsson O, Graves S, et al. Knee replacement. *Lancet*. 2012;379(9823):1331-1340.

5. Bhave A, Mont M, Tennis S, Nickey M, Starr R, Etienne G. Functional problems and treatment solutions after total hip and knee joint arthroplasty. *J Bone Joint Surg Am*. 2005;87 Suppl 2:9-21.

6. Wylde V, Dieppe P, Hewlett S, Learmonth ID. Total knee replacement: Is it really an effective procedure for all? *Knee*. 2007;14(6):417-423.

7. Beswick AD, Wylde V, Gooberman-Hill R, Blom A, Dieppe P. What proportion of patients report long-term pain after total hip or knee replacement for osteoarthritis? A systematic review of prospective studies in unselected patients. *BMJ Open*. 2012;2(1):e000435-2011-000435. Print 2012.

8. Eitner A, Hofmann GO, Schaible HG. Mechanisms of osteoarthritic pain. studies in humans and experimental models. *Front Mol Neurosci*. 2017;10:349.

9. Arendt-Nielsen L. Pain sensitisation in osteoarthritis. *Clin Exp Rheumatol*. 2017;35 Suppl 107(5):68-74.

10. Arendt-Nielsen L, Nie H, Laursen MB, et al. Sensitization in patients with painful knee osteoarthritis. *Pain*. 2010;149(3):573-581.

11. Fingleton C, Smart K, Moloney N, Fullen BM, Doody C. Pain sensitization in people with knee osteoarthritis: A systematic review and meta-analysis. *Osteoarthritis Cartilage*. 2015;23(7):1043-1056.

12. Lluch E, Torres R, Nijs J, Van Oosterwijck J. Evidence for central sensitization in patients with osteoarthritis pain: A systematic literature review. *Eur J Pain*. 2014;18(10):1367-1375.

13. Wright A, Moss P, Sloan K, et al. Abnormal quantitative sensory testing is associated with persistent pain one year after TKA. *Clin Orthop Relat Res*. 2015;473(1):246-254.

14. Schache MB, McClelland JA, Webster KE. Lower limb strength following total knee arthroplasty: A systematic review. *Knee*. 2014;21(1):12-20.

15. Anneli H, Nina SK, Arja H, et al. Effect of total knee replacement surgery and postoperative 12 month home exercise program on gait parameters. *Gait Posture*. 2017;53:92-97.

16. Bade MJ, Struessel T, Dayton M, et al. Early high-intensity versus low-intensity rehabilitation after total knee arthroplasty: A randomized controlled trial. *Arthritis Care Res (Hoboken)*. 2017;69(9):1360-1368.

17. Fransen M, Nairn L, Bridgett L, et al. Post-acute rehabilitation after total knee replacement: A multicenter randomized clinical trial comparing long-term outcomes. *Arthritis Care Res (Hoboken)*. 2017;69(2):192-200.

18. Jakobsen TL, Kehlet H, Husted H, Petersen J, Bandholm T. Early progressive strength training to enhance recovery after fast-track total knee arthroplasty: A randomized controlled trial. *Arthritis Care Res (Hoboken)*. 2014;66(12):1856-1866.

19. Piva SR, Almeida GJ, Gil AB, DiGioia AM, Helsel DL, Sowa GA. Effect of comprehensive behavioral and exercise intervention on physical function and activity participation after total knee replacement: A pilot randomized study. *Arthritis Care Res (Hoboken)*. 2017;69(12):1855-1862.

20. Pohl T, Brauner T, Wearing S, Stamer K, Horstmann T. Effects of sensorimotor training volume on recovery of sensorimotor function in patients following lower limb arthroplasty. *BMC Musculoskelet Disord*. 2015;16:195-015-0644-9.

21. Skoffer B, Maribo T, Mechlenburg I, Hansen PM, Soballe K, Dalgas U. Efficacy of preoperative progressive resistance training on postoperative outcomes in patients undergoing total knee arthroplasty. *Arthritis Care Res (Hoboken)*. 2016;68(9):1239-1251.

22. Mikkelsen LR, Mechlenburg I, Soballe K, et al. Effect of early supervised progressive resistance training compared to unsupervised home-based exercise after fast-track total hip replacement applied to patients with preoperative functional limitations. A single-blinded randomised controlled trial. *Osteoarthritis Cartilage*. 2014;22(12):2051-2058.

23. Mark-Christensen T. Genoptræningstilbuddet efter knæalloplastik. *Fysioterapeuten*. 2017;99(9).

24. Wylde V, Dennis J, Beswick AD, et al. Systematic review of management of chronic pain after surgery. *Br J Surg*. 2017;104(10):1293-1306.

25. Beswick AD, Wylde V, Gooberman-Hill R. Interventions for the prediction and management of chronic postsurgical pain after total knee replacement: Systematic review of randomised controlled trials. *BMJ Open*. 2015;5(5):e007387-2014-007387.

26. Ageberg E, Link A, Roos EM. Feasibility of neuromuscular training in patients with severe hip or knee OA: The individualized goal-based NEMEX-TJR training program. *BMC Musculoskelet Disord*. 2010;11:126-2474-11-126.

27. Skou ST, Roos EM, Laursen MB, et al. A randomized, controlled trial of total knee replacement. *N Engl J Med*. 2015;373(17):1597-1606.

28. Boudreau SA, Farina D, Falla D. The role of motor learning and neuroplasticity in designing rehabilitation approaches for musculoskeletal pain disorders. *Man Ther*. 2010;15(5):410-414.

29. Graven-Nielsen T, Arendt-Nielsen L. Impact of clinical and experimental pain on muscle strength and activity. *Curr Rheumatol Rep*. 2008;10(6):475-481.

30. Edwards PK, Mears SC, Lowry Barnes C. Preoperative education for hip and knee replacement: Never stop learning. *Curr Rev Musculoskelet Med*. 2017;10(3):356-364.

31. Louw A, Diener I, Butler DS, Puentedura EJ. Preoperative education addressing postoperative pain in total joint arthroplasty: Review of content and educational delivery methods. *Physiother Theory Pract*. 2013;29(3):175-194.

32. McDonald S, Page MJ, Beringer K, Wasiak J, Sprowson A. Preoperative education for hip or knee replacement. *Cochrane Database Syst Rev*. 2014;(5):CD003526. doi(5):CD003526.

33. Louw A, Puentedura EJ, Zimney K, Schmidt S. Know pain, know gain? A perspective on pain neuroscience education in physical therapy. *J Orthop Sports Phys Ther*. 2016;46(3):131-134.

34. Lluch E, Duenas L, Falla D, et al. Preoperative pain neuroscience education combined with knee joint mobilization for knee osteoarthritis: A randomized controlled trial. *Clin J Pain*. 2018;34(1):44-52.

35. Lluch Girbes E, Meeus M, Baert I, Nijs J. Balancing "hands-on" with "hands-off" physical therapy interventions for the treatment of central sensitization pain in osteoarthritis. *Man Ther*. 2015;20(2):349-352.

36. Ageberg E, Roos EM. Neuromuscular exercise as treatment of degenerative knee disease. *Exerc Sport Sci Rev*. 2015;43(1):14-22.

37. Nijs J, Malfliet A, Ickmans K, Baert I, Meeus M. Treatment of central sensitization in patients with 'unexplained' chronic pain: An update. *Expert Opin Pharmacother*. 2014;15(12):1671-1683.

38. Louw A, Diener I, Butler DS, Puentedura EJ. The effect of neuroscience education on pain, disability, anxiety, and stress in chronic musculoskeletal pain. *Arch Phys Med Rehabil*. 2011;92(12):2041-2056.

39. Roos EM, Roos HP, Lohmander LS, Ekdahl C, Beynnon BD. Knee injury and osteoarthritis outcome score (KOOS)--development of a self-administered outcome measure. *J Orthop Sports Phys Ther*. 1998;28(2):88-96.

40. Collins NJ, Misra D, Felson DT, Crossley KM, Roos EM. Measures of knee function: International knee documentation committee (IKDC) subjective knee evaluation form, knee injury and osteoarthritis outcome score (KOOS), knee injury and osteoarthritis outcome score physical function short form (KOOS-PS), knee outcome survey activities of daily living scale (KOS-ADL), lysholm knee scoring scale, oxford knee score (OKS), western ontario and McMaster universities osteoarthritis index (WOMAC), activity rating scale (ARS), and tegner activity score (TAS). *Arthritis Care Res (Hoboken)*. 2011;63 Suppl 11:S208-28.

41. Roos EM, Lohmander LS. The knee injury and osteoarthritis outcome score (KOOS): From joint injury to osteoarthritis. *Health Qual Life Outcomes*. 2003;1:64-7525-1-64.

42. Roos EM, Engelhart L, Ranstam J, et al. ICRS recommendation document: Patient-reported outcome instruments for use in patients with articular cartilage defects. *Cartilage*. 2011;2(2):122-136.

43. Freynhagen R, Baron R, Gockel U, Tolle TR. painDETECT: A new screening questionnaire to identify neuropathic components in patients with back pain. *Curr Med Res Opin*. 2006;22(10):1911-1920.

44. Zigmond AS, Snaith RP. The hospital anxiety and depression scale. *Acta Psychiatr Scand*. 1983;67(6):361-370.

45. Herrmann C. International experiences with the hospital anxiety and depression scale--a review of validation data and clinical results. *J Psychosom Res*. 1997;42(1):17-41.

46. Waddell G, Newton M, Henderson I, Somerville D, Main CJ. A fear-avoidance beliefs questionnaire (FABQ) and the role of fear-avoidance beliefs in chronic low back pain and disability. *Pain*. 1993;52(2):157-168.

47. Scopaz KA, Piva SR, Wisniewski S, Fitzgerald GK. Relationships of fear, anxiety, and depression with physical function in patients with knee osteoarthritis. *Arch Phys Med Rehabil*. 2009;90(11):1866-1873.

48. Sanchez K, Palazzo C, Escalas C, et al. Patient-preference disability assessment for disabling knee osteoarthritis: Validity and responsiveness of the McMaster-toronto arthritis patient preference disability questionnaire. *Ann Phys Rehabil Med*. 2016;59(4):255-262.

49. van Baar ME, Dekker J, Oostendorp RA, et al. The effectiveness of exercise therapy in patients with osteoarthritis of the hip or knee: A randomized clinical trial. *J Rheumatol*. 1998;25(12):2432-2439.

50. Kamper SJ, Ostelo RW, Knol DL, Maher CG, de Vet HC, Hancock MJ. Global perceived effect scales provided reliable assessments of health transition in people with musculoskeletal disorders, but ratings are strongly influenced by current status. *J Clin Epidemiol*. 2010;63(7):760-766.e1.

51. Sullivan MJL, Bishop SR, Pivik J. The pain catastrophizing scale: Development and validation. *Psychol Assess*. 1995;7(4):524-532.

52. Bierke S, Petersen W, Wright D, et al. Influence of anxiety and pain catastrophizing on the course of pain within the first year after uncomplicated total knee replacement: A prospective study; pain catastrophizing as a predictor for postoperative pain and opiate consumption in total joint arthroplasty patients. *Archives of orthopaedic and trauma surgery JID - 9011043 OTO - NOTNLM*.

53. Wright D, Hoang M, Sofine A, Silva JP, Schwarzkopf R. Pain catastrophizing as a predictor for postoperative pain and opiate consumption in total joint arthroplasty patients. *Arch Orthop Trauma Surg*. 2017;137(12):1623-1629.

54. Farrar JT, Young JP,Jr, LaMoreaux L, Werth JL, Poole RM. Clinical importance of changes in chronic pain intensity measured on an 11-point numerical pain rating scale. *Pain*. 2001;94(2):149-158.

55. Thompson LR, Boudreau R, Hannon MJ, et al. The knee pain map: Reliability of a method to identify knee pain location and pattern. *Arthritis Rheum*. 2009;61(6):725-731.

56. Dobson F, Hinman RS, Roos EM, et al. OARSI recommended performance-based tests to assess physical function in people diagnosed with hip or knee osteoarthritis. *Osteoarthritis Cartilage*. 2013;21(8):1042-1052.

57. Mikkelsen LR, Mikkelsen S, Soballe K, Mechlenburg I, Petersen AK. A study of the inter-rater reliability of a test battery for use in patients after total hip replacement. *Clin Rehabil*. 2015;29(2):165-174.

58. Thorborg K, Petersen J, Magnusson SP, Holmich P. Clinical assessment of hip strength using a hand-held dynamometer is reliable. *Scand J Med Sci Sports*. 2010;20(3):493-501.

59. Mentiplay BF, Perraton LG, Bower KJ, et al. Assessment of lower limb muscle strength and power using hand-held and fixed dynamometry: A reliability and validity study. *PLoS One*. 2015;10(10):e0140822.

60. Vangsgaard S, Taylor JL, Hansen EA, Madeleine P. Changes in H reflex and neuromechanical properties of the trapezius muscle after 5 weeks of eccentric training: A randomized controlled trial. *J Appl Physiol (1985)*. 2014;116(12):1623-1631.

61. U.S. Food and Drug Administration. What is a serious adverse event? https://www.fda.gov/Safety/MedWatch/HowToReport/ucm053087.htm. Updated 02.01.2016. Accessed 18.01., 2018.

62. Skou ST, Graven-Nielsen T, Rasmussen S, Simonsen OH, Laursen MB, Arendt-Nielsen L. Widespread sensitization in patients with chronic pain after revision total knee arthroplasty. *Pain*. 2013;154(9):1588-1594.

Aalborg Universitet

Statistical analysis plan for the NEPNEP trial - A randomized controlled trial for chronic pain after primary total knee arthroplasty

Larsen, Jesper Bie; Skou, Søren Thorgaard; Laursen, Mogens; Bruun, Niels Henrik; Arendt-Nielsen, Lars; Madeleine, Pascal

Publication date:
2023

Document Version
Publisher's PDF, also known as Version of record

Link to publication from Aalborg University

Citation for published version (APA):
Larsen, J. B., Skou, S. T., Laursen, M., Bruun, N. H., Arendt-Nielsen, L., & Madeleine, P. (2023, Jan 18). Statistical analysis plan for the NEPNEP trial - A randomized controlled trial for chronic pain after primary total knee arthroplasty. (1 ed.).

General rights
Copyright and moral rights for the publications made accessible in the public portal are retained by the authors and/or other copyright owners and it is a condition of accessing publications that users recognise and abide by the legal requirements associated with these rights.

- Users may download and print one copy of any publication from the public portal for the purpose of private study or research.
- You may not further distribute the material or use it for any profit-making activity or commercial gain
- You may freely distribute the URL identifying the publication in the public portal -

Take down policy
If you believe that this document breaches copyright please contact us at vbn@aub.aau.dk providing details, and we will remove access to the work immediately and investigate your claim.

Downloaded from vbn.aau.dk on: november 08, 2023

AALBORG UNIVERSITY

Statistical analysis plan for the NEPNEP trial – a randomized controlled trial for chronic pain after primary total knee arthroplasty

Larsen, Jesper Bie; Skou, Søren Thorgaard; Laursen, Mogens; Bruun, Niels Henrik; Arendt-Nielsen, Lars; Madeleine, Pascal

Publication date:
17th January 2023

Document version:
1.0

Section 1: Administrative information

Title and trial registration

Item 1a: Descriptive title

Statistical analysis plan for the NEPNEP trial – a randomized controlled trial for chronic pain after primary total knee arthroplasty.

Item 1b: Trial registration number

Trial registration: ClinicalTrials.gov: NCT03886259 (registered 22.03.2019)

Version

Item 2: Statistical analysis plan (SAP version number with dates

Statistical analysis plan version 1.0. Date: 17th January 2023

Protocol version

Item 3:

The SAP is based on the protocol approved by the North Denmark Region Committee on Health Research Ethics (N-20180046) and the study protocol which was published the 24.02.2020 (1). The SAP was made publicly available prior to the last participant has completed the 12-month follow-up and before commencing any analyses of the outcomes.

SAP revisions

Item 4a/b/c:

No revisions have been made

Roles and responsibilities

Item 5: Roles, affiliations, and SAP contributors

Principal investigator:

Jesper Bie Larsen, PT, PhD, Musculoskeletal Health and Implementation, Department of Health Science and Technology, Faculty of Medicine, Aalborg University, Aalborg, Denmark

Study chair:

Søren Thorgaard Skou, PT, PhD, Professor, Research Unit for Musculoskeletal Function and Physiotherapy, Department of Sports Science and Clinical Biomechanics, University of Southern Denmark, Odense, Denmark, and The Research Unit PROgrez, Department of Physiotherapy and Occupational Therapy, Næstved-Slagelse-Ringsted Hospitals, Region Zealand, Denmark

Mogens Laursen, PhD, Orthopedic Surgery Research Unit, Aalborg University Hospital, Aalborg, Denmark

Niels Henrik Bruun, Unit of Clinical Biostatistics, Aalborg University Hospital, Aalborg, Denmark

Lars Arendt-Nielsen, PhD, Professor, Translational Pain Biomarkers, Department of Health Science and Technology, Faculty of Medicine, Aalborg University, Aalborg, Denmark

Pascal Madeleine, PhD, Professor, Sports Sciences – Performance and Technology, Department of Health Science and Technology, Faculty of Medicine, Aalborg University, Aalborg, Denmark

Item 6a: Signature of person writing the SAP

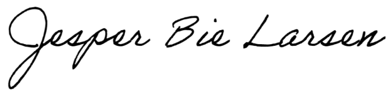

Date: 17.01.2023

Item 6b: Signature of senior statistician responsible

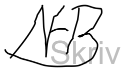

Skriv tekst her

Item 6c: Signature of chief investigator

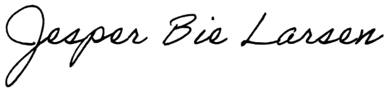

Date: 17.01.2023

<u>Section 2: Introduction</u>

Background and rationale

Item 7:

Osteoarthritis is considered the most frequent cause of disability and pain in the elderly population, and the knee joint is one of the joints most commonly and severely affected(2,3). End-stage osteoarthritis is often treated with knee arthroplasty and primary total knee arthroplasty (TKA) is considered an effective treatment for pain relief and improved function(4,5). However, several studies have reported less favorable outcomes after primary TKA(6,7). Two systematic reviews found chronic pain rates after primary TKA of 13-17% among patients 12 months post-operatively and chronic pain rates at 2-7 years post-operatively varying between 8-27%(8,9). The causes of chronic pain after TKA remains largely unexplained(10). Signs of peripheral and central sensitization has been observed in chronic pain patients following TKA(10,11). The causes of chronic pain have been shown to be complex(12) and it has been suggested that the chronic pain in patients after TKA is predominantly centrally driven(10,11).

There is a lack of evidence-based treatments available for this group of patients. Therefore, this randomized controlled trial aims at investigating whether neuromuscular exercises combined with pain neuroscience education will provide superior results in terms of pain relief and improved physical performances compared to pain neuroscience education alone at 12-months follow-up.

Objectives

Item 8: Description of objectives and hypothesis

The objective is to evaluate whether a 12-week neuromuscular exercise program combined with pain neuroscience education is superior to pain neuroscience education alone in terms of improving pain and physical performances at 12-months follow-up in a population of patients with chronic pain after primary TKA.

Hypothesis:

We hypothesize that the patients randomized to neuromuscular exercise and pain neuroscience education will improve significantly more in pain and physical performances from baseline to 12-months than the patients randomized to pain neuroscience education alone.

Section 3: Trial methods

Trial design

Item 9: Description of trial design

This trial is designed as a randomized controlled superiority trial. Treatment allocation will be in a 1:1 ratio. The patients will be randomized to receive either neuromuscular exercises and pain neuroscience education or pain neuroscience education alone(1). The primary endpoint is at 12-months follow-up and a secondary endpoint, consisting of long-term follow-up, at 24-months.

Randomization

Item 10: Randomization details

The randomization was performed using computer-generated random numbers in permuted blocks of four to eight patients. After informed consent and baseline assessment had been conducted, the principal investigator randomly assigned patients to either of the treatment arms based on the computer-generated randomization. Thereafter, the allocation was provided to the patients.

The outcome assessors are blinded to the group allocation, are not a part of the provided interventions and are not affiliated with any of the treatment sites. Patients and physiotherapists providing the interventions cannot be blinded towards randomization.

Sample size

Item 11: Full details of sample size calculation

For $KOOS_4$ and KOOS scores, a minimal clinically important difference of 10 is estimated, and commonly used(13). A sample size calculation was conducted to estimate how large a sample size is required to give the study a power of 90% to detect a minimum improvement of 10-point on the $KOOS_4$, after 12 months follow-up, in the neuromuscular exercise and pain neuroscience education

group compared with the pain neuroscience education alone group (with a standard deviation of 15)(13,14). A two-sided significance level at 0.05 was set and results revealed that 49 patients are required in both groups. To account for possible missing data and a loss to follow-up of 20%, a total of 60 patients in each group was planned to be enrolled.

However, this study was impacted by the COVID-19 pandemic making recruitment particularly difficult and causing higher dropout rate than originally anticipated. Therefore, we were not able to recruit as many participants as planned and we decided to stop the trial early after recruiting for 44 months. Therefore, a total of 71 patients have been enrolled with 36 patients in the neuromuscular exercise and pain neuroscience education group and 35 patients in the pain neuroscience education alone group.

Framework

Item 12: Description og hypothesis testing framework

All outcomes, i.e., primary, secondary, and other outcomes, will be evaluated in a superiority framework, hypothesizing that the patients receiving neuromuscular exercises and pain neuroscience education will improve more than patients receiving pain neuroscience education alone. A confidence interval including 10 points or more in the $KOOS_4$ score will be interpreted as a clinical meaningful difference, although we will interpret results with caution given the fact that we did not reach the target sample size.

Statistical interim analysis and stopping guidance

Item 13:

No interim analysis was planned, and no adjustment of significance level was made. No stopping rules was defined a priory.

Timing of final analysis

Item 14:

The analysis of primary, secondary, and other outcomes will be conducted collectively when all patients have reached the 12-month follow-up. The 12-month follow-up is expected to be finished in March 2023. An independent statistician (NHB) will conduct the analysis. Data from all time points (baseline, 3-, 6-, and 12-months) will be included in the analysis.

The analysis of the primary, pre-specified secondary and other outcomes (presented in item 26 and table 2) will be reported in the primary 12-month follow-up publication. Remaining secondary outcomes will be reported in subsequent, secondary publications.

Evaluation of long-term outcomes based on baseline to 24-months (including baseline, 3-, 6-, 12-, and 24-months) follow-up will be conducted when all patients have reached the secondary endpoint at 24-month follow-up and this data collection has ended.

Timing of outcome assessments

Item 15:

All primary, secondary, and other outcomes will be evaluated at baseline, 3-, 6-, 12-, and 24-months, except for adverse events and adherence to interventions, which will be evaluated continuously during the intervention period and at 3-months, i.e., after the interventions have stopped. Further details can be found in the published study protocol(1)

Section 4: Statistical principles:

Confidence intervals and p-values

Item 16: Level of statistical significance

All conducted statistical tests will be two-sided and will be evaluated by a significance level of 5% (i.e., p= 0.05).

Item 17: Description of planned adjustment for multiplicity

The NEPNEP trial has one clearly specified primary outcome and the secondary and other outcomes will serve as supportive and/or hypothesis generating, which is why multiplicity is not considered to be an issue (15).

Item 18: Confidence intervals

The presented confidence intervals will 95% and be two-sided.

Adherence and protocol deviation

Item 19a: Definition of adherence to interventions

Adherence will be defined as participation in both pain neuroscience education sessions (valid for both groups). For the patients receiving neuromuscular exercises, adherence will be defined as participation in minimum 75% of the neuromuscular exercise sessions (i.e., 18 out of 24 exercise sessions). Adherence is registered by the physical therapists in charge of the neuromuscular exercise sessions and the pain neuroscience education sessions.

Item 19b: Description of how adherence to interventions will be presented

Adherence, for those randomized to neuromuscular exercise and pain neuroscience education, will be reported as the numbers and percentages of patients participating in at least 18 neuromuscular exercise sessions and participating in both pain neuroscience education sessions. Adherence, for those randomized to pain neuroscience education alone, will be reported as numbers and percentages of patients participating in both pain neuroscience education sessions.

Item 19c & 19d: Definition of protocol violation for the trial and how they will be presented

It is specified as an exclusion criterion if patients experience chronic pain due to loosening of implant or prosthesis failure which requires revision surgery. However, during the follow-up, patients might be referred to revision TKA surgery, e.g., based on new available information to the surgeon. Therefore, undergoing revision TKA during follow-up is defined as a major protocol

deviation, which could impact the outcomes of the trial. Number of patients in both randomized groups will be reported.

Analysis populations

Item 20: Definition of analysis populations

All outcomes will be analyzed according to the intention-to-treat principles. The intention-to-treat population will be all subjects randomized to either group. A per-protocol analysis will be conducted based on patient adherence to interventions. Therefore, the following will be excluded from the per-protocol analysis: 1) The patients who did not participate in both pain neuroscience education sessions, 2) The patients in the neuromuscular exercises and pain neuroscience education group participating in below 75% (18 out of 24) of the neuromuscular exercise sessions and 3) patients receiving revision surgery at any time-point during follow-up.

Section 5: Trial population

Screening data

Item 21: Reporting of screening data

The recruitment period, from start date to end date, will be displayed as well as the total numbers of patients screened for eligibility throughout the recruitment period.

Eligibility

Item 22: Summary of eligibility criteria

Patients were eligible to participate in the trial if they fulfilled the inclusion criteria:

- Male or female aged 40-80 years
- Body Mass Index (BMI) between 19-40 kg/m^2
- Subjects with pain after primary TKA and ≥ 12 months post-operative
- For the index knee, duration of knee pain > 6 months.
- For the index knee, a perceived average daily pain score ≥ 4 (moderate-to-severe pain) over the last week prior to recruitment on a numerical rating scale (NRS, 0 (no pain) to 10 (maximum pain)).

Study exclusion criteria were:

- chronic pain due to loosening of implant or prosthesis failure requiring revision surgery
- secondary causes of arthritis to the knee, such as rheumatoid arthritis or sequelae from previous accidents
- surgery (including arthroscopy) of the index knee within three months prior to recruitment
- injury to the index knee within 12 months prior to visit
- acute pain, other than in the index knee, affecting the lower limb and/or trunk at the time of baseline testing
- participation in other pain trials two weeks prior to recruitment
- pregnancy
- drug and alcohol abuse

- rheumatoid arthritis, neurologic illnesses, or primary pain area other than knee (e.g., low back pain or upper extremity pain)
- lack of ability to adhere to protocol

Recruitment

Item 23 & 24: Information to be included in CONSORT flow diagram

A CONSORT flow diagram will be displayed, including the following information:

- Number of patients assessed for eligibility throughout the recruitment period
- Number of patients not meeting the inclusion criteria's or not consenting to participate
- Number of patients eligible for inclusion
- Number of patients randomized to both treatment arms
- Number of patients with follow-up assessment at 3-, 6-, 12-months for the primary analysis of 12-months follow-up[1]
- Number of patients with follow-up assessment at 3-, 6-, 12-, 24-months for the secondary long-term analysis of 24-months follow-up[1]
- Number of withdrawals or loss-to-follow-up for each timepoint and reasons for withdrawal or loss-to-follow-up
- Number of patients included in the intention-to-treat analysis and the per-protocol analysis

Baseline patient characteristics

Item 25a: List of baseline characteristics to be summarized

Patients will be described with baseline, demographic characteristics which include age, sex, height, body mass, BMI, average daily pain intensity over last week, index knee, dominant leg, time-since-TKA-surgery, TKA in non-index knee, comorbidities, and scores on the Hospital Anxiety and Depression Scale(16). The following comorbidities will be recorded: Osteoarthritis in other areas than the index knee, chronic pain from other sites than the index knee, chronic obstructive pulmonary disease, diabetes, and cardiovascular disease. Comorbidities will be registered using the medical journals.

Item 25b: Details on how baseline characteristics will be descriptively summarized

Table 1 illustrates how the baseline characteristics will be presented. Continuous data will be presented as mean and SD if data is normal distributed and as median and range if data is non-normal distributed. Categorical data will be presented as numbers and percentages. No test for statistical significance for the baseline characteristics will be conducted in line with recommendations by the CONSORT statement (17). Instead, the clinical importance of any imbalances will be considered.

[1] Number of patients with follow-up assessment is defined as patients with data available for the primary outcome ($KOOS_4$). This will be displayed for each follow-up for both randomized groups.

Table 1: Patient baseline characteristics:

Characteristics	Neuromuscular exercise and pain neuroscience education group	Pain neuroscience education alone group
Age (years), mean (SD)		
Sex (men/women, n, %)		
Height (cm), mean (SD)		
Body mass (kg), mean (SD)		
Body mass index (kg/m^2), mean (SD)		
Average daily pain intensity over last week (numerical rating scale), mean (SD)		
Index knee (left/right, n, %)		
Dominant leg (left/right, n, %)		
Time since surgery (months), mean (SD)		
Total knee arthroplasty in non-index knee (yes/no, n, %)		
Comorbidities* (n, %)		
The Hospital Anxiety and Depression Scale (0-21), mean (SD)		

* The following comorbidities will be recorded: Osteoarthritis in other areas than the index knee, chronic pain from other sites than the index knee, chronic obstructive pulmonary disease, diabetes, and cardiovascular disease.

Section 6: Analysis

Outcome definitions

Item 26: Specification of outcome and timings

Table 2 specifies which outcomes are collected, the timepoints for their assessment and the analysis methods. Further details can be found in the open access protocol (1).

Table 2: Overview of primary, secondary and other outcomes. For more details, please refer to the open access protocol (1)

	Instrument for assessment	Timing of assessment	Analysis method
**Primary outcome – reported in primary publication**			

$KOOS_4$, mean value of four KOOS subscales scores	KOOS subscales pain, symptoms, activities of daily living and knee-related quality of life<br>Each question in KOOS is assigned a score from 0 to 4 and a normalized score (100 indicating no symptoms and 0 indicating extreme symptoms) is calculated for each subscale. The $KOOS_4$ subscales scores are aggregated and averaged as the primary outcome	Baseline, 3-, 6-, 12-, and 24-months	Repeated measures mixed model
**Secondary outcomes – reported in primary publication**			
KOOS	All KOOS subscales, i.e., pain, symptoms, activity of daily living, sport/recreation and knee-related quality of life are individually reported	Baseline, 3-, 6-, 12-, and 24-months	Repeated measures mixed model
Global Perceived Effect	Questionnaire	Baseline, 3-, 6-, 12-, and 24-months	Repeated measures mixed model
40-meter fast-paced walk test	Time to complete the 40-meter walking test and calculation of walking speed (meters/second)	Baseline, 3-, 6-, 12-, and 24-months	Repeated measures mixed model
Stair climb test	Time to complete the stair climb test	Baseline, 3-, 6-, 12-, and 24-months	Repeated measures mixed model
30-second chair stand test	Number of chair stands in the 30sec. chair stand test	Baseline, 3-, 6-, 12-, and 24-months	Repeated measures mixed model
Usage of pain medication	Patient self-report of usage of pain medication during last week registered as yes/no. Registration of the number of Paracetamols (1 gram) and Ibuprofen and other non-steroidal anti-inflammatory drugs (400 mg.). If any additional pain medication was used, this will be registered as well	Baseline, 3-, 6-, 12-, and 24-months	Poisson regression model
**Other outcome – reported in primary publication**			
Adverse events	Number of adverse events self-reported by the patients and observed by the physiotherapists supervising the interventions	Continuously during the intervention period	Poisson regression model
Hospital Anxiety and Depression Scale	Questionnaire. Reported as a baseline characteristic	Baseline	No statistical analysis
Other treatments received	Patient self-report of other types of treatment received, defined as treatments that the patient had initiated because of the index knee (e.g., acupuncture, manual therapy, surgery, physiotherapy)	Baseline, 3-, 6-, 12-, and 24-months	Poisson regression model
**Secondary outcomes – reported in secondary publications**			

PainDETECT	Questionnaire	Baseline, 3-, 6-, 12-, and 24-months	Repeated measures mixed model
Fear-avoidance Beliefs Questionnaire – Physical Activity	Questionnaire	Baseline, 3-, 6-, 12-, and 24-months	Repeated measures mixed model
Pain Catastrophizing Scale	Questionnaire	Baseline, 3-, 6-, 12-, and 24-months	Repeated measures mixed model
Pain intensity in various situations	Average daily pain intensity over the last week, maximal pain intensity during rest (day and night), stair climbing, and walking using a numerical rating scale	Baseline, 3-, 6-, 12-, and 24-months	Repeated measures mixed model
Pain location	Number of painful sites concerning habitual pain areas using a pain drawing on an anatomical body chart	Baseline, 3-, 6-, 12-, and 24-months	Repeated measures mixed model
Pressure pain thresholds	Measured using a handheld algometer (Somedic, Hörby Sweden) locally at the index knee and extrasegmentally at the forearm	Baseline, 3-, 6-, 12-, and 24-months	Repeated measures mixed model
Conditioned pain modulation	Measured using pressure pain threshold as test stimuli and a spring-based pressure clamp as conditioning stimuli	Baseline, 3-, 6-, 12-, and 24-months	Repeated measures mixed model
Pinprick hyperalgesia	Measured locally at the index knee and extrasegmentally at the forearm using a pinprick nylon filament (Chicago Medical Supply)	Baseline, 3-, 6-, 12-, and 24-months	Repeated measures mixed model
Temporal summation	Measured locally at the index knee and extrasegmentally at the forearm using a pinprick nylon filament (Chicago Medical Supply)	Baseline, 3-, 6-, 12-, and 24-months	Repeated measures mixed model
Dynamic mechanic allodynia	Measured locally at the index knee and extrasegmentally at the forearm using a cotton swab	Baseline, 3-, 6-, 12-, and 24-months	Repeated measures mixed model
Deep somatic hyperalgesia	Measured locally at the index knee and extrasegmentally at the forearm using a pressure algometer (syringe)	Baseline, 3-, 6-, 12-, and 24-months	Repeated measures mixed model
Maximal leg extension power	Measured in Watt using a leg extension power rig (Nottingham power rig, Nottingham, UK) for index and non-index knee	Baseline, 3-, 6-, 12-, and 24-months	Repeated measures mixed model
Maximal isometric muscle strength of knee extensors and flexors	Measured in Newton using a handheld dynamometer (Lafayette Manual Muscle Tester, Loughborough, UK or MicroFET2, Hoggan Scientific, LLC, Salt Lake City UT, USA<br>Calculation of isometric hamstring/quadriceps (H/Q) ratio for index and non-index knee using the formula "isometric hamstring strength	Baseline, 3-, 6-, 12-, and 24-months	Repeated measures mixed model

	divided with isometric quadriceps strength = H/Q ratio”		

KOOS: Knee injury and Osteoarthritis Outcome Score.

Analysis methods

Item 27:

Primary, pre-specified secondary and other outcomes presented in table 2 will be reported in the primary 12-month follow-up publication. The same outcomes will be presented for the secondary publication of long-term follow-up when the 24-month data collection is completed (approximately March 2024). Remaining secondary outcomes presented in table 2 will be presented in subsequent, secondary publications.

The primary outcome will be the between-group change in $KOOS_4$ from baseline to 12-month follow-up. Statistical tests will be dependent on data distribution. Validation of normal distribution will be done by reviewing data frequency in histograms and tests for normality (Shapiro-Wilk). For continuous outcomes, we expect data to be normally distributed, and therefore, will be using a repeated measures mixed model with patients as random effect and visit (baseline, 3-, 6-, 12-months) and treatment arm (neuromuscular exercises and pain neuroscience education or pain neuroscience education alone) as fixed effects, and with adjustment for baseline imbalance. Interaction between follow-up and treatment arm was also included in the models. Both crude and adjusted values will be reported.

Secondary outcomes will be analyzed like the primary outcome as well as the long-term (24-month) follow-up analysis. Frequency of adverse events and other types of treatment received (see table 2) will be compared between-groups at the 12-months follow-up using a Poisson regression model with a robust error variance(18). Similarly, between-group comparison of relative risks concerning usage of pain medication will be analyzed using a Poisson regression model, with robust error variance.

A confidence interval including 10 points or more for the primary outcome $KOOS_4$ will be interpreted as a clinical meaningful difference.

A responder analysis, illustrating the proportion of patients in each randomized group that experienced a minimal clinically important improvement (i.e., minimum improvement of 10 points) for the primary outcome $KOOS_4$, will be made to evaluate between-group difference baseline to 12-months follow-up. Results will be analyzed using a Chi-squared test.

A figure including data from all time points (baseline, 3-, 6-, and 12-months) will be presented to visualize the mean value and 95% CI over time in $KOOS_4$ for the patients randomized to either neuromuscular exercise and pain neuroscience education or pain neuroscience education alone. A similar figure will be displayed for the secondary, long-term (24-months) follow-up analysis including all time points (baseline, 3-, 6-, 12-, and 24-months)

The patient’s individual trajectory of pain (illustrated by the KOOS pain subscale) and physical performance (illustrated by the 40-meter fast-paced walk test) will be depicted in graphs for both

randomized groups in the subsequent, secondary 12-months follow-up publications and for the secondary long-term (24-month) follow-up publication.

Missing data:

Item 28:

Since the linear mixed effects models includes all patients when at least the baseline value or a follow-up value is present, no imputation will be required(19,20). Number of data points available in each group at baseline, 3-, 6-, and 12-months will be displayed in primary and secondary publications. Number of data points available in each group at baseline, 3-, 6-, 12-, and 24-months will be displayed in in the secondary long-term follow-up (24-months) publication.

Additional analyses

Item 29:

Exploratory analyses of associations between pre-specified outcomes are planned and will be reported in secondary publications.

Multivariate linear regression models based on the enter method with an adjustment for age, sex, and BMI will be conducted to analyze associations between the primary outcome ($KOOS_4$) and pain-related outcomes, bedside quantitative sensory testing outcomes and physical performance outcomes. Two regression models will be conducted. One will include $KOOS_4$ as the dependent variable and leg extension power and maximal isometric muscle strength for knee flexors and extensor as independent variables and one will include $KOOS_4$ as the dependent variable and pressure pain thresholds, temporal summation, the fear-avoidance beliefs questionnaire and the pain catastrophizing questionnaire as independent variables.

An exploratory analysis is planned and will be reported in a secondary publication. All patients will be stratified according to their conditioned pain modulation responses, i.e., classified as a conditioned pain modulation responder, a conditioned pain modulation non-responder or no change in conditioned pain modulation. Further details for the stratification are described in the study *Larsen et al. Stratification of facilitatory or inhibitory conditioned pain modulation responses in patients with chronic knee pain. Explorative analysis from a multicenter trial (under review at European Journal of Pain).* Following stratification, data will be analyzed like the primary analysis (item 27). This analysis will allow us to verify if the treatment effect for the primary and secondary outcomes at 12-months is associated with conditioned pain modulation responses. Results will be presented with a figure including all data points (baseline, 3-, 6-, and 12-months) to visualize the mean and 95% CI over time for the primary outcomes $KOOS_4$ for each randomized group, stratified according to the conditioned pain modulation responses.

Further exploratory analyses can be conducted if deemed relevant.

Harms

Item 30: Sufficient detail provided on summarizing harms

Adverse events that may have occurred during the trial period will be identified by the patients (self-reported) and by the physiotherapists supervising the interventions (observations) (see table 3).

Adverse events are characterized as occurring in either the index knee or sites other than the index knee and serious events are defined according to the definitions from the U.S. Food and Drug Administration(21). Adverse events will be descriptively summarized for each randomized group similar to table 3 and be reported in the primary 12-months follow-up publication.

Table 3: Adverse events. The table will include all serious and non-serious adverse events that were registered during the 12-month follow-up period. Serious adverse events associated with the interventions is defined as events that result in death, a life-threating condition, hospitalization, disability or permanent damage, or other serious events, that does not fit the other outcomes (21).

**Adverse events**	**Neuromuscular exercise and pain neuroscience education group**	**Pain neuroscience education alone group**
	Number of events	
**Serious events**		
Site other than index knee:		
XXXX		
XXXX		
XXXX		
Index knee:		
XXXX		
XXXX		
XXXX		
All serious events		
**Non-serious events***		
Sites other than index knee		
XXXX		
XXXX		
XXXX		
Index knee		
XXXX		
XXXX		
XXXX		
All non-serious		

events		

* Non-serious adverse events could be, but not limited to, increased pain in index knee, swelling of index knee, decreased range of motion, distortion of joints, musculoskeletal pain.

Statistical software

Item 31: Details of statistical package used for the analysis

The statistical package StataCorp. 2021. Stata Statistical Software: Release 17. College Station, TX: StataCorp LLC was used for data management and analysis. The packages basetable and matrixtools were also used.

References

Item 32: Data management

The project is approved by The Danish Data Protection Agency (Aalborg University, 2018-899/10-0166). Data are stored in accordance with the stipulations in The Danish Personal Data Protection Act and other relevant Danish legislation. Data was recorded in hard copy during the outcome assessments and thereafter noted in Excel spreadsheets. Data entry and coding of the non-personal information will be administered by trained staff from Aalborg University. The main data set will not contain any personal information. No personal information will be shared outside the study group. All data, including hard copy data from the individual outcome assessment of the patients, will be stored securely.

The analyses described in this SAP will be the basis of all primary and secondary endpoints. Analyses will be made by the same independent statistician. The principal investigator will code the randomized groups in "group A" and "group B" before submitting the dataset to the statistician. Thereby, analyses will be blinded towards treatment allocation. First, the dataset will be provided for the statistician without information of adherence or adverse events to avoid that blinding is broken. Following finalization and reporting of the intention-to-treat analysis, the statistician will be given information on adherence and adverse events to conduct the per-protocol analysis.

To avoid the risk of misleading interpretation, the blinded results from the intention-to-treat analysis (group A vs. group B) will be presented to all authors. The author group will then decide on two different interpretations of the results, one in which group A refer to neuromuscular exercises and pain neuroscience education, and one in which group A refer to pain neuroscience education alone. The interpretations will be registered in a document titled "NEPNEP trial: Blinded data analyses statement of interpretation". Following written registration and agreeing that no further changes will be made, the randomization code is broken, and the correct interpretation can be chosen(22).

7. Reference list

1. Larsen JB, Skou ST, Arendt-Nielsen L, Simonsen O, Madeleine P. Neuromuscular exercise and pain neuroscience education compared with pain neuroscience education alone in patients with chronic

pain after primary total knee arthroplasty: study protocol for the NEPNEP randomized controlled trial. Trials. 2020 Feb 24;21:218. https://doi.org/10.1186/s13063-020-4126-5

2. Peat G, McCarney R, Croft P. Knee pain and osteoarthritis in older adults: a review of community burden and current use of primary health care. Ann Rheum Dis. 2001 Feb;60(2):91–7.

3. Dieppe PA, Lohmander LS. Pathogenesis and management of pain in osteoarthritis. Lancet. 2005;365(9463):965–73.

4. Hunter DJ, Bierma-Zeinstra S. Osteoarthritis. Lancet. 2019 Apr 27;393(10182):1745–59.

5. Price AJ, Alvand A, Troelsen A, Katz JN, Hooper G, Gray A, et al. Knee replacement. Lancet. 2018 Nov 3;392(10158):1672–82.

6. Wylde V, Beswick A, Bruce J, Blom A, Howells N, Gooberman-Hill R. Chronic pain after total knee arthroplasty. EFORT Open Rev. 2018 Aug 16;3(8):461–70.

7. Rice DA, Kluger MT, McNair PJ, Lewis GN, Somogyi AA, Borotkanics R, et al. Persistent postoperative pain after total knee arthroplasty: a prospective cohort study of potential risk factors. Br J Anaesth. 2018 Oct;121(4):804–12.

8. Beswick AD, Wylde V, Gooberman-Hill R, Blom A, Dieppe P. What proportion of patients report long-term pain after total hip or knee replacement for osteoarthritis? A systematic review of prospective studies in unselected patients. BMJ Open. 2012 Feb 22;2(1):e000435.

9. Wylde V, Dieppe P, Hewlett S, Learmonth ID. Total knee replacement: is it really an effective procedure for all? Knee. 2007 Dec;14(6):417–23.

10. Wright A, Moss P, Sloan K, Beaver RJ, Pedersen JB, Vehof G, et al. Abnormal quantitative sensory testing is associated with persistent pain one year after TKA. Clin Orthop Relat Res. 2015 Jan;473(1):246–54.

11. Phillips JR, Hopwood B, Stroud R, Dieppe PA, Toms AD. The characterisation of unexplained pain after knee replacement. Br J Pain. 2017 Nov;11(4):203–9.

12. Eitner A, Hofmann GO, Schaible HG. Mechanisms of Osteoarthritic Pain. Studies in Humans and Experimental Models. Front Mol Neurosci. 2017 Nov 3;10:349.

13. Roos EM, Lohmander LS. The Knee injury and Osteoarthritis Outcome Score (KOOS): from joint injury to osteoarthritis. Health Qual Life Outcomes. 2003 Nov 3;1:64.

14. Skou ST, Roos EM, Laursen MB, Rathleff MS, Arendt-Nielsen L, Simonsen O, et al. A Randomized, Controlled Trial of Total Knee Replacement. N Engl J Med. 2015 Oct 22;373(17):1597–606.

15. The European Agency for the Evaluation of Medicinal Products C. Points to consideron multiplicity issues in clinical trials. EMEA. 2002;

16. Zigmond AS, Snaith RP. The hospital anxiety and depression scale. Acta Psychiatr Scand. 1983 Jun;67(6):361–70.

17. Moher D, Hopewell S, Schulz KF, Montori V, Gotzsche PC, Devereaux PJ, et al. CONSORT 2010 explanation and elaboration: updated guidelines for reporting parallel group randomised trials. BMJ. 2010 Mar 23;340:c869.

18. Zou G. A modified poisson regression approach to prospective studies with binary data. Am J Epidemiol. 2004 Apr 1;159(7):702–6.

19. Ranstam J, Turkiewicz A, Boonen S, van Meirhaeghe J, Bastian L, Wardlaw D. Alternative analyses for handling incomplete follow-up in the intention-to-treat analysis: the randomized controlled trial of balloon kyphoplasty versus non-surgical care for vertebral compression fracture (FREE). BMC Med Res Methodol. 2012 Mar 24;12:35.

20. Twisk JW, Rijnhart JJ, Hoekstra T, Schuster NA, ter Wee MM, Heymans MW. Intention-to-treat analysis when only a baseline value is available. Contemp Clin Trials Commun [Internet]. 2020 Dec 1 [cited 2023 Jan 10];20. Available from: https://pubmed.ncbi.nlm.nih.gov/33319119/

21. US Food and DA. What is a serious adverse event? [Internet]. Vol. 2018. 2016. Available from: https://www.fda.gov/Safety/MedWatch/HowToReport/ucm053087.htm

22. Järvinen TLN, Sihvonen R, Bhandari M, Sprague S, Malmivaara A, Paavola M, et al. Blinded interpretation of study results can feasibly and effectively diminish interpretation bias. J Clin Epidemiol [Internet]. 2014 Jul 1 [cited 2022 Dec 7];67(7):769–72. Available from: http://www.jclinepi.com/article/S0895435613004861/fulltext
