# Supplementary material for: Exercise and Pain Neuroscience Education for Patients With Chronic Pain After Total Knee Arthroplasty: A Randomized Clinical Trial
Source: JAMA Netw Open. 2024 May 24;7(5):e2412179. doi: 10.1001/jamanetworkopen.2024.12179 (PMC11127128; doi:10.1001/jamanetworkopen.2024.12179)
Supplement: Supplement 2. — eAppendix 1. CONSORT Checklist for Randomized Trials eAppendix 2. TIDieR Checklist for Information to Include When Describing an Intervention eAppendix 3. CERT Checklist for What to Include When Reporting Exercise Programs eMethods 1. Pain Neuroscience Education Session 1 eMethods 2. Pain Neuroscience Education Session 2 eTable 1. Patient Baseline Characteristics for Those Attending the 12-Month Follow-up Assessment and Those who Did Not Attend eTable 2. Intention-to-Treat Analysis for Risk Ratios for Usage of Pain Medication From Baseline to 12 Months eTable 3. Per-Protocol Analysis for the Primary and Secondary Outcomes for Change From Baseline to 12 Months eTable 4. Per-Protocol Analysis for the Changes in Usage of Pain Medication From Baseline to 12 Months [file jamanetwopen-e2412179-s002.pdf]

Supplemental Online Content

Larsen JB, Skou ST, Laursen M, Bruun NH, Arendt-Nielsen L, Madeleine P. Exercise and pain neuroscience education for patients with chronic pain after total knee arthroplasty: a randomized clinical trial. *JAMA Netw Open*. 2024;7(5):e2412179. doi:10.1001/jamanetworkopen.2024.12179

eAppendix 1. CONSORT Checklist for Randomized Trials
eAppendix 2. TIDieR Checklist for Information to Include When Describing an Intervention
eAppendix 3. CERT Checklist for What to Include When Reporting Exercise Programs
eMethods 1. Pain Neuroscience Education Session 1
eMethods 2. Pain Neuroscience Education Session 2
eTable 1. Patient Baseline Characteristics for Those Attending the 12-Month Follow-up Assessment and Those who Did Not Attend
eTable 2. Intention-to-Treat Analysis for Risk Ratios for Usage of Pain Medication From Baseline to 12 Months
eTable 3. Per-Protocol Analysis for the Primary and Secondary Outcomes for Change From Baseline to 12 Months
eTable 4. Per-Protocol Analysis for the Changes in Usage of Pain Medication From Baseline to 12 Months

This supplemental material has been provided by the authors to give readers additional information about their work.

© 2024 Larsen JB et al. *JAMA Network Open*.

Reporting checklist for randomised trial Based on the CONSORT guidelines.

Instructions to authors

Complete this checklist by entering the page numbers from your manuscript where readers will find each of the items listed below.

Your article may not currently address all the items on the checklist. Please modify your text to include the missing information. If you are certain that an item does not apply, please write "n/a" and provide a short explanation.

Upload your completed checklist as an extra file when you submit to a journal.

In your methods section, say that you used the CONSORTreporting guidelines, and cite them as:

Schulz KF, Altman DG, Moher D, for the CONSORT Group. CONSORT 2010 Statement: updated guidelines for reporting parallel group randomised trials

		Reporting Item	Page Number
**Title and Abstract**			
Title	#1a	Identification as a randomized trial in the title.	Title
Abstract	#1b	Structured summary of trial design, methods, results, and conclusions	Abstract
**Introduction**			
Background and objectives	#2a	Scientific background and explanation of rationale	Introduction
Background and objectives	#2b	Specific objectives or hypothesis	Introduction
**Methods**			
Trial design	#3a	Description of trial design (such as parallel, factorial) including allocation ratio.	Study design
Trial design	#3b	Important changes to methods after trial commencement (such as eligibility criteria), with reasons	NA
Participants	#4a	Eligibility criteria for participants	Participants<br>Participants
Participants	#4b	Settings and locations where the data were collected	Interventions
Interventions	#5	The experimental and control interventions for each group with sufficient details to allow replication, including how and when they were actually administered	Outcomes
Outcomes	#6a	Completely defined prespecified primary and secondary outcome measures, including how and when they were assessed	

© 2024 Larsen JB et al. *JAMA Network Open*.

Outcomes	#6b	Any changes to trial outcomes after the trial commenced, with reasons	NA
Sample size	#7a	How sample size was determined.	Sample size calculation
Sample size	#7b	When applicable, explanation of any interim analyses and stopping guidelines	NA
Randomization – Sequence generation	#8a	Method used to generate the random allocation sequence.	Randomization and masking
Randomization - Sequence generation	#8b	Type of randomization; details of any restriction (such as blocking and block size)	Randomization and masking
Randomization - Allocation concealment mechanism	#9	Mechanism used to implement the random allocation sequence (such as sequentially numbered containers), describing any steps taken to conceal the sequence until interventions were assigned	Randomization and masking
Randomization - Implementation	#10	Who generated the allocation sequence, who enrolled participants, and who assigned participants to interventions	Randomization and masking
Blinding	#11a	If done, who was blinded after assignment to interventions (for example, participants, care providers, those assessing outcomes) and how.	Randomization and masking
Blinding	#11b	If relevant, description of the similarity of interventions	NA
Statistical methods	#12a	Statistical methods used to compare groups for primary and secondary outcomes	Data analysis
Statistical methods	#12b	Methods for additional analyses, such as subgroup analyses and adjusted analyses	Data analysis
**Results**			
Participant flow diagram (strongly recommended)	#13a	For each group, the numbers of participants who were randomly assigned, received intended treatment, and were analysed for the primary outcome	Results and Figure 1
Participant flow	#13b	For each group, losses and exclusions after randomization, together with reason	Figure 1
Recruitment	#14a	Dates defining the periods of recruitment and follow-up	Results
Recruitment	#14b	Why the trial ended or was stopped	Sample size calculation
Baseline data	#15	A table showing baseline demographic and clinical characteristics for each group	Table 1

© 2024 Larsen JB et al. *JAMA Network Open*.

Numbers analysed	#16	For each group, number of participants (denominator) included in each analysis and whether the analysis was by original assigned groups	Table 2 and eTable 1
Outcomes and estimation	#17a	For each primary and secondary outcome, results for each group, and the estimated effect size and its precision (such as 95% confidence interval)	Results and Table 2 and eTable 2
Outcomes and estimation	#17b	For binary outcomes, presentation of both absolute and relative effect sizes is recommended	eTable 2
Ancillary analyses	#18	Results of any other analyses performed, including subgroup analyses and adjusted analyses, distinguishing pre-specified from exploratory	NA
Harms	#19	All important harms or unintended effects in each group (For specific guidance see CONSORT for harms)	Results
**Discussion**			
Limitations	#20	Trial limitations, addressing sources of potential bias, imprecision, and, if relevant, multiplicity of analyses	Discussion
Generalisability	#21	Generalisability (external validity, applicability) of the trial findings	Discussion
Interpretation	#22	Interpretation consistent with results, balancing benefits and harms, and considering other relevant evidence	Discussion
**Other information**			
Registration	#23	Registration number and name of trial registry	Study design
Protocol	#24	Where the full trial protocol can be accessed, if available	Study design
Funding	#25	Sources of funding and other support (such as supply of drugs), role of funders	Funding acknowledgement

© 2024 Larsen JB et al. *JAMA Network Open*.

The TIDieR (Template for Intervention Description and Replication) Checklist*:

Information to include when describing an intervention and the location of the information

Item Number	Primary paper (page or appendix number)	Where located **	Other † (details)
	**BRIEF NAME**		
**1.**	Provide the name or a phrase that describes the intervention.	Interventions –	eMethod 1 and 2
	**WHY**		
**2.**	Describe any rationale, theory, or goal of the elements essential to the intervention.	Introduction and Interventions	eMethod 1 and 2
	**WHAT**		
**3.**	Materials: Describe any physical or informational materials used in the intervention, including those provided to participants or used in intervention delivery or in training of intervention providers. Provide information on where the materials can be accessed (e.g. online appendix, URL).	Interventions	eMethod 1 and 2
**4.**	Procedures: Describe each of the procedures, activities, and/or processes used in the intervention, including any enabling or support activities.	Interventions	eMethod 1 and 2
	**WHO PROVIDED**		
**5.**	For each category of intervention provider (e.g. psychologist, nursing assistant), describe their expertise, background and any specific training given.	Interventions	
	**HOW**		
**6.**	Describe the modes of delivery (e.g. face-to-face or by some other mechanism, such as internet or telephone) of the intervention and whether it was provided individually or in a group.	Interventions	

© 2024 Larsen JB et al. *JAMA Network Open*.

**7.**	**WHERE**<br>Describe the type(s) of location(s) where the intervention occurred, including any necessary infrastructure or relevant features.	Participants	
	**WHEN and HOW MUCH**		
**8.**	Describe the number of times the intervention was delivered and over what period of time including the number of sessions, their schedule, and their duration, intensity or dose.	Interventions	
	**TAILORING**		
**9.**	If the intervention was planned to be personalised, titrated or adapted, then describe what, why, when, and how.	Interventions	
	**MODIFICATIONS**	NA	
**10.‡**	If the intervention was modified during the course of the study, describe the changes (what, why, when, and how).		
	**HOW WELL**		
**11.**	Planned: If intervention adherence or fidelity was assessed, describe how and by whom, and if any strategies were used to maintain or improve fidelity, describe them.	Data analysis	
**12.‡**	Actual: If intervention adherence or fidelity was assessed, describe the extent to which the intervention was delivered as planned.	Results	

** **Authors** - use N/A if an item is not applicable for the intervention being described. **Reviewers** – use '?' if information about the element is not reported/not sufficiently reported.

† If the information is not provided in the primary paper, give details of where this information is available. This may include locations such as a published protocol or other published papers (provide citation details) or a website (provide the URL).

‡ If completing the TIDieR checklist for a protocol, these items are not relevant to the protocol and cannot be described until the study is complete.

* We strongly recommend using this checklist in conjunction with the TIDieR guide (see *BMJ* 2014;348:g1687) which contains an explanation and elaboration for each item.

* The focus of TIDieR is on reporting details of the intervention elements (and where relevant, comparison elements) of a study. Other elements and methodological features of studies are covered by other reporting statements and checklists and have not been duplicated as part of the TIDieR checklist. When a **randomised trial** is being reported, the TIDieR checklist should be used in conjunction with the CONSORT statement (see www.consort-statement.org) as an extension of **Item 5 of the CONSORT 2010 Statement.** When a **clinical trial protocol** is being reported, the TIDieR checklist should be used in conjunction with the SPIRIT statement as an extension of **Item 11 of the SPIRIT 2013 Statement** (see www.spirit-statement.org). For alternate study designs, TIDieR can be used in conjunction with the appropriate checklist for that study design (see www.equator-network.org).

© 2024 Larsen JB et al. *JAMA Network Open*.

Consensus on Exercise Reporting Template

A Checklist for what to include when reporting exercise programs

Section/ Topic	Item #	Checklist item	Location**	
			Primary paper (page, table, appendix)	† Other (paper or protocol, website (URL)
**WHAT: materials**	1	Detailed description of the type of exercise equipment (e.g. weights, exercise equipment such as machines, treadmill, bicycle ergometer etc)	Interventions	Open access protocol
**WHO: provider**	2	Detailed description of the qualifications, teaching/supervising expertise, and/or training undertaken by the exercise instructor	Intervention	
**HOW: delivery**	3	Describe whether exercises are performed individually or in a group	Interventions	Open access protocol
	4	Describe whether exercises are supervised or unsupervised and how they are delivered	Interventions	Open access protocol
	5	Detailed description of how adherence to exercise is measured and reported	Data analysis	Open access protocol
	6	Detailed description of motivation strategies	NA	
	7a	Detailed description of the decision rule(s) for determining exercise progression	Interventions	Open access protocol
	7b	Detailed description of how the exercise program was progressed		Open access protocol
	8	Detailed description of each exercise to enable replication (e.g. photographs, illustrations , video etc)	NA	Original paper, reference 16
	9	Detailed description of any home program component (e.g. other exercises, stretching etc)	NA	
	10	Describe whether there are any non-exercise components (e.g. education, cognitive behavioural therapy, massage etc)	Interventions	Open access protocol

© 2024 Larsen JB et al. *JAMA Network Open*.

	11	Describe the type and number of adverse events that occurred during exercise	Results	Original paper, reference 16
**WHERE: location**	12	Describe the setting in which the exercises are performed	Patients	
**WHEN, HOW MUCH: dosage**	13	Detailed description of the exercise intervention including, but not limited to, number of exercise repetitions/sets/sessions, session duration, intervention/program duration etc		
**TAILORING: what, how**	14a	Describe whether the exercises are generic (one size fits all) or tailored whether tailored to the individual	Interventions	Open access protocol
	14b	Detailed description of how exercises are tailored to the individual	Interventions	Open access protocol
	15	Describe the decision rule for determining the starting level at which people commence an exercise program (such as beginner, intermediate, advanced etc)	Interventions	Open access protocol
**HOW WELL: planned, actual**	16a	Describe how adherence or fidelity to the exercise intervention is assessed/measured	Results	
	16b	Describe the extent to which the intervention was delivered as planned	NA	

***It is recommended that this checklist is used in conjunction with the Explanation and Elaboration Statement which is a guide each item in the CERT Checklist**

The CERT Checklist is designed for reporting details of an exercise intervention. The CERT Checklist should be used in conjunction with a reporting checklist appropriate for the study type e.g. the CONSORT Statement (www.consort-statement.org) for randomised controlled trials, the SPIRIT Statement (www.spirit-statement.org) for a clinical trial protocol. For further guidance regarding reporting guidelines please consult the EQUATOR network (www.equator-network.org)

****** Authors – please use N/A if an item is not applicable Reviewers – please use ”?” if information is not provided or not/insufficiently reported

† If the information is not provided in the primary paper that is under consideration, please provide details of where this information is available e.g. in a published protocol, published papers (provide citation details) or on a website (provide the URL).

© 2024 Larsen JB et al. *JAMA Network Open*.

AALBORG UNIVERSITY HOSPITAL

AALBORG UNIVERSITY
DENMARK

eMethods 1. Pain Neuroscience Education Session 1

Why does chronic pain occur after total knee replacement?

Pain education, understanding and treatment

First session

The Danish
Rheumatism Association

© 2024 Larsen JB et al. *JAMA Network Open*.

Chronic pain after total knee replacement?

20% experience chronic pain after total knee replacement

15% of these will experience severe pain

Definition of chronic pain:

- Duration >3 months

© 2024 Larsen JB et al. *JAMA Network Open*.

What is pain?

Acute pain:

- Is a personal experience
- Is ”created” inside the nervous system
- Is a natural part of life
- Is a warning signal that informs us of potentially damaging events

© 2024 Larsen JB et al. *JAMA Network Open*.

Pain is our warning system

- Nerve cells located in the body send ”danger” signals towards the brain in case of possible damaging events.
- The brain ”decides” if these signals are interpreted as dangerous and induces the pain experience.
- Pain is a normal response for an event that the brain considers as potentially dangerous for the body.
- The pain experience is induced to make us stop the damaging event or remove ourselves from the danger.

© 2024 Larsen JB et al. *JAMA Network Open*.

Pain continued…

- If the brain does not consider the situation as dangerous = No experienced pain
- Pain intensity does not necessarily depend on the amount of tissue damage.
- It is possible to experience pain without tissue damage
- The pain experience is real and not something we make up

© 2024 Larsen JB et al. *JAMA Network Open*.

Factors behind the acute pain experience

- Pain is a unique, personal experience and other persons will not experience the same pain

- The acute pain experience is influenced by multiple factors, e.g.:
    - Tissue damage
    - Context and surroundings
    - Sex
    - Culture
    - Previous experiences with pain

© 2024 Larsen JB et al. *JAMA Network Open*.

Chronic pain

- The brain has concluded you are in ”danger” and needs to be warned

- Multiple factors influence the chronic pain experience
  - Personal factors, e.g., sex, age, social and family relations, financial situations
  - Psychological factors, e.g., catastrophizing thoughts, anxiety or fear of pain, depression, stress
  - Lack of coping strategies when in pain
  - Localized joint damage or inflammation, altered biomechanics and changed activation of muscles
  - Systemic inflammation
  - Sleep
  - **Sensitization**

© 2024 Larsen JB et al. *JAMA Network Open*.

Sensitization – what is it?

- The communication between the nerve cells is more efficient than normal
- Lack of inhibition of the pain signals

- The nervous system is plastic and can change and adapt

© 2024 Larsen JB et al. *JAMA Network Open*.

Sensitization – what is it?

Consequence:

- A painful stimulus will be experienced as more painful than normally
- A normally non-painful stimuli will induce a pain experience

© 2024 Larsen JB et al. *JAMA Network Open*.

Sensitization – what is it?

1) The pain experience is still there despite tissue healing has occurred

2) Pain starts to spread into other areas of the body

3) The pain intensity increases

4) Multiple body movements induces pain – even small body movements

5) The pain becomes more unpredictable – it becomes difficult to figure out what causes the pain and when it arises

6) The pain experience can become more and more connected to thoughts and emotions

7) The pain experience can become more connected to previous painful experiences – expectations alone can induce pain

© 2024 Larsen JB et al. *JAMA Network Open*.

Pain and tissue damage

- Pain primarily associated with tissue damage

- Pain primarily associated with the nervous system

© 2024 Larsen JB et al. *JAMA Network Open*.

Influencing factors for chronic pain

- Personal factors, e.g., sex, age, social and family relations, financial situations
- Psychological factors, e.g., catastrophizing thoughts, anxiety or fear of pain, depression, stress
- Lack of coping strategies when in pain
- Localized joint damage or inflammation, altered biomechanics and changed activation of muscles
- Systemic inflammation
- Sleep
- Sensitization

© 2024 Larsen JB et al. *JAMA Network Open*.

Behavior, thoughts, and reactions

Thoughts and perception are nerve signals and can therefore be involved in ”creating” danger signals that could lead to or maintain pain, e.g.,

- ”When I experience pain, it is because I am getting worse”
- ”I am not giving my tissue enough time to heal”
- ”My pain will never get better”

© 2024 Larsen JB et al. *JAMA Network Open*.

Behavior, thoughts, and reactions

- Poor or lack of pain coping skills
- Poor or lack of sleep
- Stress

© 2024 Larsen JB et al. *JAMA Network Open*.

Chronic pain and physical activity

- Chronic pain is not a sign of rest being required. The chronic pain experience is not ”dangerous”, but the brain perceives ”danger” and induces the pain to warn you to stop your activity.

- ”It is not safe for me to be physical active”
- ”I am having difficulties in enjoying the things I used to enjoy doing”

© 2024 Larsen JB et al. *JAMA Network Open*.

How can we treat chronic pain?

Through our newly gained insight into chronic pain, we can affect our thoughts and perceptions, e.g.,

- ”When I experience pain, it does not necessary mean that I am damaging myself”
- Understand that it is not ”dangerous” to move and be physical active
- Meditation, breathing-exercises or yoga can be used to remove thoughts focusing on the pain experience
- Do something that makes you feel good

© 2024 Larsen JB et al. *JAMA Network Open*.

How can we treat chronic pain?

- Ensure proper sleep
- Diminish stress
- Improved knowledge of chronic pain provides an opportunity to better understand and manage your own situation
- Make sure you control the pain and not the other way around, e.g.,
  - Find the right balance between activities that you know will induce pain and resting
  - Understand that it is not ”dangerous” to move

© 2024 Larsen JB et al. *JAMA Network Open*.

Active or passive strategies?

Active coping strategies:

- Investigate and understand the background for the pain problem
- Find alternative ways to move and doing your activities of daily living
- Gently push the limits for the pain experience without doing too much or doing too little
- Stay positive
- Make small, achievable goals that you work towards
- Be patient
- Accept relapses

© 2024 Larsen JB et al. *JAMA Network Open*.

Active or passive strategies?

Passive coping strategies:

- Avoid any activities and movements that are painful
- Do nothing
- Wait for something to change
- Waiting for the right person to change something for you (*the right person is you!)*

© 2024 Larsen JB et al. *JAMA Network Open*.

Home-assignment

Graded-exposure for an activity where you would normally experience pain:

Start by finding an activity that you would like to do more often.

What is your baseline – how much of this activity can you do without experiencing a pain flare up?

Start at this limit and slowly increase the amount of activity – one small step at the time.

Be patient! Do not give up or panic if/when a pain flare up is experienced.

© 2024 Larsen JB et al. *JAMA Network Open*.

This research project is initiated and supported by

AALBORG UNIVERSITY HOSPITAL

AALBORG UNIVERSITY
DENMARK

Lions Danmark

The Svend Andersen Foundation

The Danish
Rheumatism Association

© 2024 Larsen JB et al. *JAMA Network Open*.

AALBORG UNIVERSITY HOSPITAL

AALBORG UNIVERSITY DENMARK

eMethods 2. Pain Neuroscience Education Session 2

Why does chronic pain occur after total knee replacement?

Pain education, understanding and treatment

Second session

The Danish Rheumatism Association

© 2024 Larsen JB et al. *JAMA Network Open*.

Summary from first session

- Pain is a warning signal that informs us of potentially damaging events → the brain has ”decided” that you are in ”danger” and needs to be alerted
- Chronic pain is typically a sign of the brain interpreting you as being in ”danger” – it is rarely a sign of you damaging yourself
- Pain intensity does not necessarily depend on the amount of tissue damage
- The pain experience is real and not something we make up

© 2024 Larsen JB et al. *JAMA Network Open*.

- Sensitization: The nervous system´s influence on the pain

© 2024 Larsen JB et al. *JAMA Network Open*.

The many factors behind chronic pain

- Personal factors, e.g., sex, age, social and family relations, financial situations
- Psychological factors, e.g., catastrophizing thoughts, anxiety or fear of pain, depression, stress
- Lack of coping strategies when in pain
- Localized joint damage or inflammation, altered biomechanics and changed activation of muscles
- Systemic inflammation
- Sleep
- Sensitization

© 2024 Larsen JB et al. *JAMA Network Open*.

What can make the brain induce pain?

”Danger signals” versus ”safety signals”

- Mainly presence of ”danger” signals can induce pain
- Mainly presence of ”safety” signals promote that less, or no pain is experienced

© 2024 Larsen JB et al. *JAMA Network Open*.

Overview of “danger”- and “safety” signals

“Danger” signals – promote pain	“Safety” signals – decrease pain
Fear of movement	Have a good ”coach” – possible yourself
Poor physical fitness	Being physical fit and mobile
Lack of knowledge concerning pain	Have knowledge on pain
”I have joint degenerative changes”	Good friends and social relations
Negative attitude	Positive attitude
Hopelessness	Plans, hopes and inspirations
Low expectations	Patience and persistence
Poor nutrition	Healthy and varied nutrition
Poor sleep	Good and adequate sleep

© 2024 Larsen JB et al. *JAMA Network Open*.

Your own pain medication

- The brain can produce hormones
- Some home hormones are pain-relieving
- Your access to your own pain-relieving medication is affected by the “danger”- and “safety” signals

- *Conclusion*: You are in charge for the access to your own pain-relieving medication

© 2024 Larsen JB et al. *JAMA Network Open*.

How can we treat chronic pain?

Retrain the brain – ”Plasticity”

- **The purpose** is to train the brain and nervous system back to protecting you when it is necessary and not when it is unnecessary.
- **Understanding** is very important for chronic pain. If you can change your understanding of the pain, you can change your perception of the pain experience**.**
- With this **knowledge**, your self-confidence to move will increase – and thereby the possibility to adjust your limits little by little.

© 2024 Larsen JB et al. *JAMA Network Open*.

Plasticity

- Find the right balance when pushing your limits to initiate changes and adaptions without enhancing the "danger" signals.
- The longer your brain and nervous system has been (over)protecting you, they better it becomes at it.
- Plasticity of the brain is part of what gives you chronic pain – and plasticity is what can help you reduce the chronic pain.

© 2024 Larsen JB et al. *JAMA Network Open*.

Be your own ”coach”

- Move a little more than you would normally do
- Do activities that get your pulse up
- To gradually increase your activities is the best way to train your brain and nervous system to be less (over)protective

Remember that pain is there to protect you and not necessarily a sign of you damaging yourself!

© 2024 Larsen JB et al. *JAMA Network Open*.

Your own experiences – ”home assignment” from first session

Which activities did you try to increase/improve?

Was it possible to establish a limit and to increase the amount of your activity from that point?

How did it go? Did it work?

Did you experience a flare up in pain along the way?

© 2024 Larsen JB et al. *JAMA Network Open*.

Recipe for adapting the brain towards an activity

- **Activity**: Walking in the forest
- **Aim**: Would like to be able to walk for an hour
- **How much activity** is required before **a major pain flare up is present**: 30 min.
- **How much activity** is required before the pain experience is present: 5 min.
- **Plan**: Initiate your activity by walking between 5-30 min. – depending on what you feel you can manage
- **Evaluate** along the way. Adjust when necessary, e.g., increase activity when you feel comfortable at the present level

© 2024 Larsen JB et al. *JAMA Network Open*.

Summary

- Many different factors can influence that we develop chronic pain and the chronic pain experience

- Thoughts can be part of creating “danger” signals which again can be part of inducing pain

- Important to get good and adequate sleep, reduce stress and be physical active to reduce chronic pain

© 2024 Larsen JB et al. *JAMA Network Open*.

Summary continued

- There is not a single exercise, exercise type or specific movement that is better than others = Choose to do something that makes you feel good and comfortable

- Meditation, yoga and breathing-exercises can for some be useful to manage chronic pain

© 2024 Larsen JB et al. *JAMA Network Open*.

Sticky note

- The pain experience is real and not something we make up
- Chronic pain is rarely a sign of you damaging yourself
- Mainly presence of ”danger” signals can induce pain
- Mainly presence of ”safety” signals promote that less, or no pain is experienced

- The brain is plastic and can be trained into being less (over)protective

Remember that pain is there to protect you and not necessarily a sign of you damaging yourself!

© 2024 Larsen JB et al. *JAMA Network Open*.

This research project is initiated and supported by

AALBORG UNIVERSITY HOSPITAL

AALBORG UNIVERSITY
DENMARK

Lions Danmark

The Svend Andersen Foundation

The Danish
Rheumatism Association

© 2024 Larsen JB et al. *JAMA Network Open*.

eTable 1. Patient Baseline Characteristics for Those Attending the 12-Month Follow-up Assessment and Those who Did Not Attend

	**Attended 12-month follow-up (n=46)**	**Did not attend 12-months follow-up (N=23)**
Age (years), median (IQR)	67.2 (62.1 to 70.9)	67.2 (59.9 to 73.3)
Sex (females and males, n, %)	26 (57%)<br>20 (43%)	14 (61%)<br>9 (39%)
Height (cm), median (IQR)	1.7 (1.6 to 1.8)	1.7 (1.6 to 1.7)
Body mass (kg), median (IQR)	95.0 (80.7 to 107.3)	94.8 (74.7 to 105.0)
Body mass index (kg/m^2), median (IQR)	33.3 (29.7 to 36.0)	31.7 (25.9 to 36.3)
Average daily pain intensity over last week (NRS), median (IQR)	5.0 (4.0 to 6.0)	5.0 (5.0 to 7.0)
Index knee (right, n, %)	20 (44)	13 (57)
Dominant leg (right, n, %)	39 (85)	21 (96)
Time since surgery (years), median (IQR)	2.4 (1.7 to 4.2)	3.4 (1.8 to 4.9)
Have a total knee arthroplasty in non-index knee (n, %)	13 (28)	5 (22)
Patients with one or more comorbidities* (n, %)	37 (80)	22 (96)
Distribution of comorbidities (n, %)		
Osteoarthritis in other joints than the index knee	25 (54%)	13 (57%)
Chronic pain from other sites than the index knee	24 (52%)	17 (74%)
Chronic obstructive pulmonary disease	4 (9%)	2 (9%)
Diabetes	5 (11%)	3 (13%)
Cardiovascular disease	4 (13%)	4 (17%)
The Hospital Anxiety and Depression Scale – Anxiety total score (0-21), median (IQR)	8.0 (4.0 to 9.0)	8.0 (3.0 to 10.0)
The Hospital Anxiety and Depression Scale – Depression total score (0-21), median (IQR)	12.5 (5.8 to 15.0)	13.0 (4.0 to 15.0)

© 2024 Larsen JB et al. *JAMA Network Open*.

IQR: Interquartile range. NRS: Numerical rating scale. * The following comorbidities were recorded: Osteoarthritis in other joints than the index knee, chronic pain from other sites than the index knee, chronic obstructive pulmonary disease, diabetes, and cardiovascular disease. Scores from the Hospital Anxiety and Depression Scale are interpreted as 0-7 as “no case”, 8-10 “borderline case”, 11 and above “case” (Zigmond, 1983).

© 2024 Larsen JB et al. *JAMA Network Open*.

eTable 2. Intention-to-Treat Analysis for Risk Ratios for Usage of Pain Medication From Baseline to 12 Months

	Usage of pain medication at 12-months	
Outcome (number of data points$_{\text{neuromuscular exercise and PNE group}}$, number of data points$_{\text{PNE alone group}}$)*	Neuromuscular exercise and pain neuroscience education group (95% CI)	Pain neuroscience education alone group (95% CI)
Proportion of users of pain medication		
Baseline	0.75 (0.62 to 0.91)	0.82 (0.70 to 0.96)
12-months (110, 106)	0.75 (0.59 to 0.95)	0.73 (0.56 to 0.94)
Risk ratio for usage of pain medication at 12-months vs. baseline.	1.00 (0.80 to 1.25)	0.89 (0.65 to 1.21)
Risk ratio for usage of pain medication at 12-months in the neuromuscular exercise and pain neuroscience education group vs. pain neuroscience education alone group		
Model 1	1.03 (0.73 to 1.46, P=0.86)	
Model 2	1.02 (0.73 to 1.43, P=0.92)	

* There were 144 possible data points for the neuromuscular exercises and PNE group (36 at baseline, 3, 6, and 12-months) and 136 possible data points for the PNE alone group (33 at baseline, 3, 6, and 12-months). CI: Confidence interval. Model 1 adjusted for patient, follow-up, treatment arm and interaction between follow-up and treatment arm and model 2 further including adjustment for age, sex, and body mass index.

© 2024 Larsen JB et al. *JAMA Network Open*.

eTable 3. Per-Protocol Analysis for the Primary and Secondary Outcomes for Change From Baseline to 12 Months

Included patients adhered to participation in both pain neuroscience education sessions (valid for both groups) and for the patients receiving neuromuscular exercises, those adhering a minimum of 75% of the neuromuscular exercise sessions (i.e., 18 out of 24 exercise sessions).

Outcome (number of data points$_{\text{neuromuscular exercise and PNE group}}$, number of data points$_{\text{PNE alone group}}$)*	Improvement in neuromuscular exercise and PNE group (95% CI)	Improvement in PNE alone group (95% CI)	Between-group difference (model 1) (95% CI)	Between-group difference (model 2) (95% CI)	P-values for between-group difference (model 2)
	**Primary outcome**				
Mean change in KOOS$_4$ from baseline to 12-months (87, 92)	7.06 (2.11 to 12.01)	8.88 (4.41 to 13.34)	-1.82 (-8.49 to 4.85)	-2.05 (-9.11 to 5.00)	0.57
	**Secondary outcomes**				
KOOS Pain (87, 92)	5.15 (0.01 to 10.29)	10.18 (5.62 to 14.75)	-5.04 (-11.91 to 1.84)	-5.79 (-13.23 to 1.65)	0.13
KOOS Symptoms (87, 92)	7.84 (-0.09 to 15.78)	6.85 (-0.03 to 13.73)	1.00 (-9.51 to 11.50)	0.33 (-10.74 to 11.40)	0.95
KOOS Activities of daily living (86, 90)	3.39 (-1.25 to 8.03)	8.64 (2.20 to 15.08)	-5.25 (-13.19 to 2.69)	-5.39 (-13.25 to 2.47)	0.18
KOOS Sport/Recreation (87, 91)	5.67 (0.05 to 11.29)	10.56 (3.50 to 17.62)	-4.89 (-13.92 to 4.13)	-5.48 (-14.30 to 3.35)	0.22
KOOS Quality of life (87, 91)	9.88 (2.76 to 17.01)	10.92 (4.47 to 17.37)	-1.03 (-10.65 to 8.58)	-0.01 (-9.51 to 9.48)	0.99
Global Perceived effect (64, 65)	3.12 (2.52 to 3.73)	2.82 (2.22 to 3.43)	0.30 (-0.56 to 1.16)	0.37 (-0.52 to 1.26)	0.42
Time to walk 40 meter (87, 92)	-3.15 (-6.15 to -0.15)	-2.26 (-5.53 to 1.01)	-0.90 (-5.34 to 3.54)	-0.52 (-5.25 to 4.21)	0.83
Stair climbs (sec) (87, 92)	-2.24 (-4.82 to 0.33)	-2.26 (-3.99 to -0.52)	0.01 (-3.10 to 3.12)	0.07 (-3.15 to 3.30)	0.96
30sec. chair stand (reps.) (87, 92)	0.93 (0.21 to 1.65)	1.96 (0.97 to 2.95)	-1.03 (-2.26 to 0.20)	-1.17 (-2.41 to 0.07)	0.06

* There were 92 possible data points for the neuromuscular exercises and PNE group (23 at baseline, 3, 6, and 12-months) and 104 possible data points for the PNE alone group (26 at baseline, 3, 6, and 12-months), except for global perceived effect which had 69 possible data points for the neuromuscular exercise and PNE group (23 at 3, 6, and 12-months) and 78 possible data points for the PNE alone group (26 at 3, 6, and 12-months). PNE: Pain neuroscience education. KOOS: Knee injury and Osteoarthritis Outcome Score. CI: Confidence intervals. Reps: Repetitions. Model 1 adjusted for patient, follow-up, treatment arm and interaction between follow-up and treatment arm and model 2 further including adjustment for age, sex, and body mass index.

eTable 4. Per-Protocol Analysis for the Changes in Usage of Pain Medication From Baseline to 12 Months

Included patients adhered to participation in both pain neuroscience education sessions (valid for both groups) and for the patients receiving neuromuscular exercises, those adhering a minimum of 75% of the neuromuscular exercise sessions (i.e., 18 out of 24 exercise sessions).

	**Usage of pain medication at 12-months**	
Outcome (number of data points$_{\text{neuromuscular exercise and PNE group}}$, number of data points$_{\text{PNE alone group}}$)*	Neuromuscular exercise and pain neuroscience education group (95% CI)	Pain neuroscience education alone group (95% CI)
**Proportion of users of pain medication**		
Baseline	0.70 (0.53 to 0.91)	0.77 (0.62 to 0.95)
12-months (87, 92)	0.75 (0.58 to 0.97)	0.74 (0.56 to 0.97)
Risk ratio for usage of pain medication at 12-months vs. baseline.	1.08 (0.85 to 1.37)	0.96 (0.68 to 1.36)
**Risk ratio for usage of pain medication at 12-months in the neuromuscular exercise and pain neuroscience education group vs. pain neuroscience education alone group**		
Model 1	1.02 (0.70 to 1.48, P=0.93)	
Model 2	0.98 (0.68 to 1.42, P=0.93)	

* There were 92 possible data points for the neuromuscular exercises and PNE group (23 at baseline, 3, 6, and 12-months) and 104 possible data points for the PNE alone group (26 at baseline, 3, 6, and 12-months). Model 1 adjusted for patient, follow-up, treatment arm and interaction between follow-up and treatment arm and model 2 further including adjustment for age, sex, and body mass index.

© 2024 Larsen JB et al. *JAMA Network Open*.
