# Supplementary material for: Exercise and Pain Neuroscience Education for Patients With Chronic Pain After Total Knee Arthroplasty: A Randomized Clinical Trial
Source: JAMA Netw Open. 2024 May 24;7(5):e2412179. doi: 10.1001/jamanetworkopen.2024.12179 (PMC11127128; doi:10.1001/jamanetworkopen.2024.12179)
Supplement: Supplement 3. — Data Sharing Statement [file jamanetwopen-e2412179-s003.pdf]

Data Sharing Statement

Larsen. Exercise and Pain Neuroscience Education for Patients With Chronic Pain After Total Knee Arthroplasty. *JAMA Netw Open*. Published May 24, 2024.
doi:10.1001/jamanetworkopen.2024.12179

Data
Data available: Yes
Data types: Deidentified participant data
How to access data: Deidentified participant data and a data dictionary are available from the first author on reasonable request and after approval by the study publication committee. Data cannot be reused unless a col-laboration agreement has been signed by both parties.
When available: With publication

Supporting Documents
Document types: Statistical/analytic code, Other (please specify)
Additional Information: Online open access protocol:
https://trialsjournal.biomedcentral.com/articles/10.1186/s13063-020-4126-5 Online open access blinded interpretation: https://vbn.aau.dk/da/publications/blinded-interpretation-for-the-primary-and-secondary-outcomes-for
How to access documents: Online open access statistical analysis plan:
https://vbn.aau.dk/da/publications/statistical-analysis-plan-for-the-nepnep-trial-a-randomized-contr
When available: With publication

Additional Information
Who can access the data: Deidentified participant data and a data dictionary are available from the first author on reasonable request and after approval by the study publication committee. Data cannot be reused unless a col-laboration agreement has been signed by both parties.
Types of analyses: For research purposes
Mechanisms of data availability: After approval of proposal and with a signed data access agreement
